# Distinct lactate utilization strategies drive niche differentiation between two co-existing *Megasphaera* species in the rumen microbiome

**DOI:** 10.1093/ismejo/wraf147

**Published:** 2025-07-14

**Authors:** Cameron R Strachan, Connor M Bowers, Byung-Chul Kim, Tea Movsesijan, Viktoria Neubauer, Anna J Mueller, Xiaoqian A Yu, Fátima C Pereira, Veronika Nagl, Johannes Faas, Martin Wagner, Qendrim Zebeli, Paul J Weimer, Pieter Candry, Martin F Polz, Christopher E Lawson, Evelyne Selberherr

**Affiliations:** Centre for Food Science and Veterinary Public Health, Clinical Department for Farm Animals and Food System Science, University of Veterinary Medicine Vienna, Veterinärplatz 1, Vienna 1210, Austria; FFoQSI GmbH - Austrian Competence Centre for Feed and Food Quality, Safety and Innovation, Technopark 1D, Tulln 3430, Austria; Department of Chemical Engineering & Applied Chemistry, University of Toronto, Toronto, ON M5T 3E5, Canada; Department of Chemical Engineering & Applied Chemistry, University of Toronto, Toronto, ON M5T 3E5, Canada; Centre for Food Science and Veterinary Public Health, Clinical Department for Farm Animals and Food System Science, University of Veterinary Medicine Vienna, Veterinärplatz 1, Vienna 1210, Austria; FFoQSI GmbH - Austrian Competence Centre for Feed and Food Quality, Safety and Innovation, Technopark 1D, Tulln 3430, Austria; Centre for Food Science and Veterinary Public Health, Clinical Department for Farm Animals and Food System Science, University of Veterinary Medicine Vienna, Veterinärplatz 1, Vienna 1210, Austria; FFoQSI GmbH - Austrian Competence Centre for Feed and Food Quality, Safety and Innovation, Technopark 1D, Tulln 3430, Austria; Division of Microbial Ecology, Centre for Microbiology and Environmental Systems Science, University of Vienna, Djerassiplatz 1, Vienna 1030, Austria; Division of Microbial Ecology, Centre for Microbiology and Environmental Systems Science, University of Vienna, Djerassiplatz 1, Vienna 1030, Austria; Division of Microbial Ecology, Centre for Microbiology and Environmental Systems Science, University of Vienna, Djerassiplatz 1, Vienna 1030, Austria; School of Biological Sciences, University of Southampton, Southampton, United Kingdom; Dsm-Firmenich, Animal Nutrition and Health R&D Center, Tulln, Technopark 1, Tulln 3430, Austria; Dsm-Firmenich, Animal Nutrition and Health R&D Center, Tulln, Technopark 1, Tulln 3430, Austria; Centre for Food Science and Veterinary Public Health, Clinical Department for Farm Animals and Food System Science, University of Veterinary Medicine Vienna, Veterinärplatz 1, Vienna 1210, Austria; FFoQSI GmbH - Austrian Competence Centre for Feed and Food Quality, Safety and Innovation, Technopark 1D, Tulln 3430, Austria; Centre for Animal Nutrition and Welfare, Clinical Department for Farm Animals and Safety of Food Systems, University of Veterinary Medicine Vienna, Veterinärplatz 1, Vienna 1210, Austria; Christian Doppler Laboratory for Innovative Gut Health Concepts of Livestock, Veterinärplatz 1, Vienna 1210, Austria; Department of Bacteriology, University of Wisconsin, Madison, WI 53706, United States; Laboratory of Systems and Synthetic Biology, Wageningen University & Research, Stippeneng 4, Wageningen, 6703 HB, the Netherlands; Division of Microbial Ecology, Centre for Microbiology and Environmental Systems Science, University of Vienna, Djerassiplatz 1, Vienna 1030, Austria; Department of Chemical Engineering & Applied Chemistry, University of Toronto, Toronto, ON M5T 3E5, Canada; Centre for Food Science and Veterinary Public Health, Clinical Department for Farm Animals and Food System Science, University of Veterinary Medicine Vienna, Veterinärplatz 1, Vienna 1210, Austria

**Keywords:** lactate utilization, niche differentiation, metabolic trade-offs, *Megasphaera*, rumen

## Abstract

Lactate utilization mitigates rumen acidosis and is associated with decreased methane production in the rumen. While several lactate utilization pathways exist across different microbial species in the rumen, how they are metabolically differentiated remains unclear. Here, we show that the key lactate-utilizing species *Megasphaera hexanoica* and *Megasphaera elsdenii* display distinct growth strategies based on their fermentative end products. This allows them to co-exist and play distinct metabolic roles, which appear particularly relevant in the early stages of rumen development, as both species are highly enriched in the calf. Specifically, *M. hexanoica* is more strongly associated with rumen microbiome states that involve increased lactate utilization and preferentially runs reverse beta-oxidation (termed chain elongation) to produce butyrate and medium-chain fatty acids from lactate. As *M. elsdenii* instead utilizes lactate via the acrylate pathway to produce propionate, we leverage Enzyme Cost Minimization to predict how this pathway relates to a distinct growth strategy. We find that *M. elsdenii* maximizes growth rate when lactate transiently accumulates, which contrasts *M. hexanoica’s* invariably high-yield strategy. This trade-off, which is supported by the analysis of growth kinetics, metabolic flux, and bioreactors simulating the rumen microbiome, ultimately contributes to co-existence on lactate and may have driven niche differentiation. Lastly, we demonstrate how lactate utilization in the *Megasphaera* is threatened by toxins widespread in feed, which points to dietary interventions to support calf health.

## Introduction

Microbiome function in domestic ruminants is tightly linked to both their health and environmental impact [1–3]. While we are only beginning to understand these links mechanistically, ruminal lactate utilization is often a crucial factor. This is particularly true for cattle that are fed diets rich in readily fermentable carbohydrates, as this risks the rapid accumulation of lactic acid in the rumen. The accompanying disease is known as acute ruminal acidosis and is a particular problem for feedlot cattle [4]. A less severe form of the disease is known as sub-acute ruminal acidosis and is more common in dairy cattle [4–8]. Both acute and sub-acute ruminal acidosis are associated with a sustained depression in rumen pH and can be mitigated by lactate-utilizing bacteria, particularly those producing propionate or butyrate [9, 10]. Similarly, cow microbiomes that are feed efficient and emit low amounts of methane are also enriched with lactate utilizers, partially because lactate utilization can participate in a metabolic cascade that diverts electrons away from methanogenesis and towards organic acids that can be absorbed by the host [11–13]. However, lactate rarely accumulates in a healthy adult rumen, especially when the animal is fed a diet that is high in forage [14, 15], which is consistent with the long-term adaptation of ruminants to grasslands [16]. In contrast, lactate frequently reaches relatively high concentrations in the calf rumen, which requires rapid fermentation to support early development via the supply of short and medium-chain fatty acids (SCFA and MCFA) [17]. In nature, this process is promoted by maternal milk, which is gradually replaced commercially with high-energy milk replacers and starter feeds that lead to a decreased pH in the developing rumen [18–20]. Consistent with this decrease in pH, recent research indicates that rumen acidosis is readily inducible in the calf, with severe consequences for its health [21]. Further, such rumen perturbations during early development may impact microbiome assembly and thus have long-lasting impacts on rumen function [22]. In this context, another often overlooked perturbation to the rumen with the potential to affect lactate-utilizing microbes during periods of pH depression is the presence of common feed contaminants known as mycotoxins [23–25].

Lactate utilization pathways in the rumen microbiome include anaerobic respiration with nitrate or fumarate as electron acceptors. However, there has been substantial applied interest in pathways that directly lead to SCFAs, such as the succinate and acrylate pathways. Much of this interest stems from the fact that SCFAs supply a significant fraction of the host animals’ energy budget [26]. Additionally, the conversion of lactate to more weakly acidic SCFAs is thought to stabilize rumen pH [4]. A long-standing model organism for studying the utilization of lactate and concomitant SCFA production is *Megasphaera elsdenii*, which was isolated in the 1960s [27]. *M. elsdenii* gained further attention when it was shown to preferentially consume lactate in the presence of sugars, which involves the expression of a lactate racemase and the acrylate pathway, leading to the production of propionate [28, 29]. *M. elsdenii* also harbors the reverse-beta oxidation (rBOX) pathway and thus the potential to convert lactate to butyrate and medium-chain fatty acids (MCFAs), which, like propionate, can be absorbed by the host [30, 31]. However, butyrate and MCFA production by *M. elsdenii* has only been observed during the consumption of sugars *in vitro* [28]. When lactate is converted to butyrate or MCFAs *in vivo* remains an open question and has implications for understanding overall rumen metabolism and rumen acidosis, as butyrate and MCFA production incorporate a proton into the fermentative end product [11, 32]. Additionally, the rBOX pathway is of interest regarding the mitigation of ruminant methane emissions as it directs electrons to VFAs (rather than CO_2_ in methanogenesis), which, despite their low reduction potential [33], are one of the few alternative electron acceptors in the reductant-rich ruminal habitat [11, 34]. Recently, a strain likewise belonging to the *Megasphaera* genus, *Megasphaera hexanoica* [35], was isolated that can utilize lactate while running rBOX, a process often referred to as lactate-driven chain elongation [32, 36, 37]. By comparing *M. hexanoica* with *M. elsdenii*, we seek to first determine when lactate-driven chain elongation plays a distinct role in the rumen with respect to the acrylate pathway.

Because lactate is a key microbial resource whose concentration fluctuates in the rumen, we hypothesized that despite their shared ability to utilize lactate, the two *Megasphaera* species specialize on differences in resource availability [38]. Theory predicts that such specialization is driven by trade-offs that minimize competition over time and lead to distinct growth strategies and niches [38–40]. Understanding the degree of specialization and the underlying metabolic pathways will enable us to identify the conditions under which lactate-utilizing microbes play unique functional roles. Here, we screen metagenomes from the rumen for organisms with the potential to run the acrylate pathway or chain elongate from lactate. We find that most are enriched in the calf rumen, including *M. elsdenii* and *M. hexanoica*, which both harbor low genome-wide diversity. Supporting their co-existence on lactate, *M. hexanoica* carries out extensive chain elongation and grows slowly on lactate compared to *M. elsdenii*, which runs the acrylate pathway to enable a high-rate, lower-yield strategy during transient lactate accumulation. We then show that *M. hexanoica* has the potential to persistently promote rumen stability via lactate-drive chain elongation in the calf, where the pH is low. However, we also find that *M. hexanoica* is inhibited by mycotoxins, which are stable at low pH and prevalent in feeds that induce pH depression [41]. Taken together, we provide an example of how studying related, co-existing microbial populations that utilize a key resource provides insight into distinct functional roles and niches in the rumen microbiome.

## Materials and methods

### Analysis of publicly available sequence data

**Figure 1:**
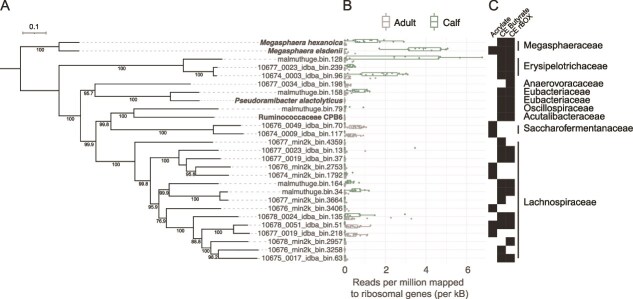
Metagenomic screen for rumen MAGs with the potential to run the acrylate pathway or lactate-driven chain elongation. (A) A concatenated marker tree of bacteria harboring the acrylate pathway or lactate-driven chain elongation. The final alignment contained 5036 positions at the amino acid level, including gaps. The maximum-likelihood tree was built with the LG model and bootstrap values over 75 are shown. The rumen MAGs were obtained from two studies (Stewart *et al. 2019* and Malmuthuge *et al.* 2019, n = 20 744 for total MAGs). Isolate genomes (shown in bold) were included from four bacteria known to chain elongate (CE) and utilize lactate. MAGs and genomes were dereplicated together at 98% ANI, which is why genomes rather than MAGs represent the two rumen *Megasphaera*. (B) The average number of reads mapped at over 98% ID to genes encoding ribosome subunits from metagenome from the adult and calf rumen (n = 117 and n = 18 for metagenomes from Stewart *et al.* 2019 and Malmuthuge *et al.*, 2019, respectively). (C) The family-level classification according to the Genome Taxonomy Database is shown on the right-hand side of the plot.

**Figure 2:**
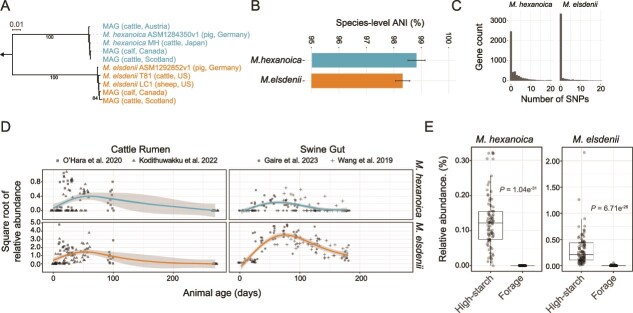
Genotypic structure and *in vivo* dynamics of *Megasphaera elsdenii* and *Megasphaera hexanoica*. (A) A concatenated-marker tree that includes *Megasphaera* strain genomes and MAGs from cows and pigs sampled across the world. The final alignment contained 2804 positions at the amino acid level and any gaps were masked. The maximum-likelihood tree was built with the LG model and bootstrap values over 75 are shown. The tree was rooted (indicated by the arrow) using a *Dialister* sp. MAG from the rumen as an outgroup (Stewart *et al. 2019*). (B) The species-level ANI was calculated pairwise between the genomes and MAGs in panel A. The mean of the pairwise comparisons is shown and the error bars represent the standard deviation. (C) The distribution of total single nucleotide polymorphisms (SNPs) in genes detected in the four metagenomes with sufficient coverage that were sampled from calves from the same herd (n = 4, Malmuthuge *et al.* 2019). (D) The relative abundance of both *Megasphaera* species over the animal’s age (days of life) based on amplicon sequence data from multiple studies of the cattle rumen and swine gut. The square root of relative abundance and a smoothed line, based on a generalized additive model (GAM) fit, and the shaded band represents the 95% confidence interval. The square root of relative abundance is shown to improve visualization of data points close to 0. (E) The relative abundance of *M. hexanoica* and *M. elsdenii* in the cattle rumen based on a re-analysis of McGovern *et al.* 2020, which compared different diets. This study collected a large amount of amplicon data that was split into high-starch and forage groups (n = 79 and n = 71, respectively). The *P* values were obtained from a Wilcoxon rank-sum test with Benjamini-Hochberg adjustment using centered log ratio transformed counts (pseudocount = 1) to mitigate the effect of compositionality.

**Figure 3:**
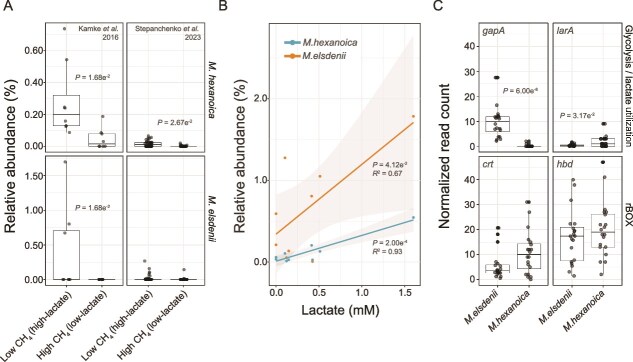
Individual *Megasphaera* ssp. associations with microbiome states that involve lactate utilization and expression of specific catabolic pathways. (A) The relative abundance of *M. elsdenii* and *M. hexanoica* in sheep (Kamke *et al.* 2016, n = 8 for each group) and cows (Stephanchenko *et al.* 2023, n = 39 and n = 32 animals for the low and high methane groups, respectively). This is based on the re-analysis of the data grouped by methane emission, where low methane-emitting ruminants also have higher amounts of lactate in both studies. The *P* values were obtained from a Wilcoxon rank-sum test with Benjamini-Hochberg adjustment using centered log ratio transformed counts (pseudocount = 1) to mitigate the effect of compositionality. (B) The correlation (Pearson) between the amount of lactate in the rumen in calves that are eight weeks of age with *M. hexanoica* and *M. elsdenii* in data from Dill-McFarland *et al.* 2017. The *P* values were obtained from t-test on the correlation coefficients. The shaded band represents the 95% confidence interval. (C) The expression levels of select genes in the rumen liquid of calves based on a re-analysis of the transcriptome of Park *et al.* 2022 (n = 20). The *gapA* (glyceraldehyde-3-phosphate dehydrogenase) gene is a well-established marker of glycolysis across taxa, whereas *larA* (lactate racemase) has been shown to be expressed during lactate utilization in *M. elsdenii*. The *crt* (enoyl-CoA hydratase) and *hbd* (3-hydroxyacyl-CoA dehydrogenase) genes are sequential reactions in rBOX. The number of reads mapped was normalized by the mean fold change in the expression of genes between the two species (*M. elsdenii* over *M. hexanoica*) to be able to compare relative expression levels. The *P* values were obtained from a Wilcoxon rank-sum test with Benjamini-Hochberg adjustment using centered log ratio transformed counts (pseudocount = 1) to mitigate the effect of compositionality.

All sequence data analysis is described in detail in the Supplementary Materials and Methods. In short, we first compiled a database of metagenomes-assembled genomes (MAGs) from the rumen to assess the distribution of lactate utilization pathways (Fig. 1). Isolate genomes from known lactate utilizers were also included as controls. To screen for the metabolic potential to run the acrylate pathway and lactate-driven chain elongation, we compiled a set of marker proteins to annotate the different pathways. These included the lactate racemase as a marker of lactate utilization and the enzymes conducting both reaction steps of the acrylate pathway. For chain elongation, enzymes involved in two key reactions for butyrate production and three reactions from rBOX were also included. MAGs and genomes harboring the potential for the acrylate pathway or lactate-driven chain elongation were then quantified by mapping metagenomic reads from the calf and adult rumen to genes encoding for ribosomal proteins. To assess the *in vivo* dynamics of the *Megasphaera* (Fig. 2D and E, Fig. 3A and B), amplicon sequence data were compiled from several studies, processed using the qiime2 environment (v. 2021.4.0) [42], and assigned to *M. hexanoica* or *M. elsdenii* using blastn (v.2.5.0+) against reference genomes (rumen strains MH and T81, respectively). The same reference genomes were used to map metatranscriptomic reads from the calf rumen (Fig. 3C and Supplementary Fig. S2B).

### Time series of batch cultures and high-throughput growth kinetics

**Figure 4:**
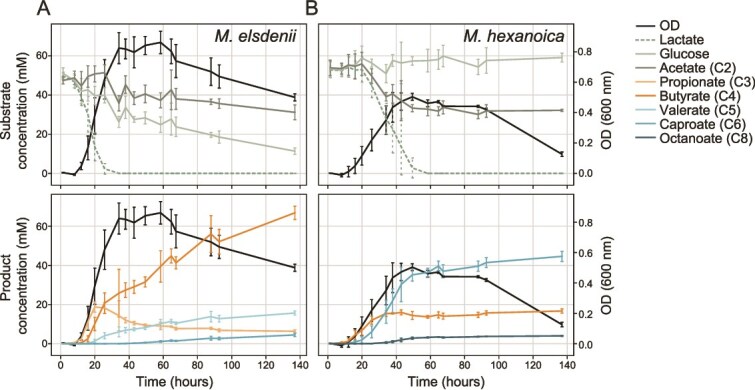
Growth and fermentation kinetics of *M. elsdenii* (A) and *M. hexanoica* (B) cultured with lactate and glucose as electron donors. The top two panels show the growth and substrate (lactate, glucose, and acetate) consumption dynamics. The line representing lactate concentrations is dashed, and acetate was included in the experiment as it is a co-substrate of chain elongation. The bottom two panels present volatile fatty acids (C2-C6, C8) production dynamics. Optical density (OD) measurements are shown on the right-hand side y-axis in all panels. The mean is shown and the error bars represent the standard deviation (n = 3 biological replicates).

Representative strains of *M. elsdenii* (DSM20460) and *M. hexanoica* (DSM106893) were used in the main batch culture growth experiments, which were grown anaerobically and sampled over time with a syringe. For example, this was done for experiments where the media contained lactate and glucose as the main electron donors (Fig. 4), and the final media pH for these experiments was measured. The same strains were also used in high-throughput kinetic characterization of microbial growth (Supplementary Fig. S3B), where the media contained 18 different lactate concentrations ranging from 0 mM to 150 mM in 96 well microplates. Further details for all growth experiments and calculation of maximum specific growth rate can be found in the Supplementary Materials and Methods.

### Enzyme Cost Minimization

**Figure 5:**
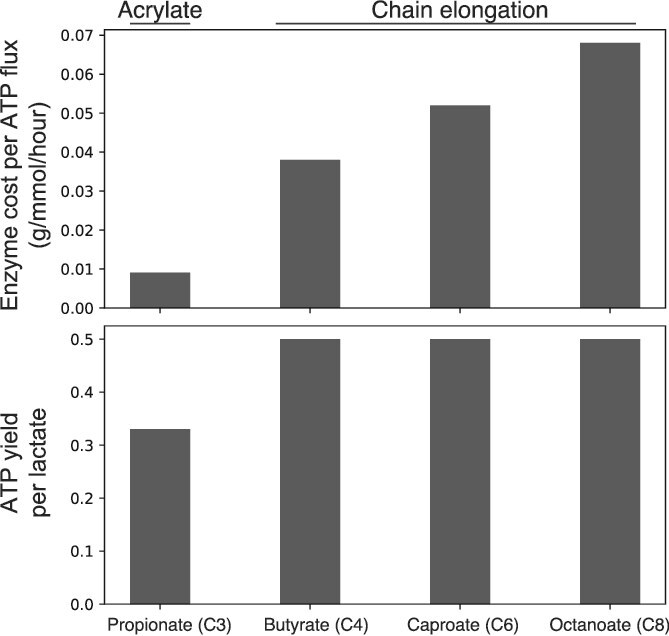
Enzyme cost minimization analysis. The production of propionate via the acrylate pathway was compared to the production of butyrate, caproate, and octanoate via chain elongation (see Materials and methods). The top and bottom panels show the predicted relative enzyme cost per ATP flux and ATP yield per lactate, respectively.

Enzyme Cost Minimization (ECM) was developed previously [43] and performed using eQuilibrator Python packages (Fig. 5, https://gitlab.com/equilibrator). A metabolic pathway is first constructed by defining the substrates, products and stoichiometry of constituent reactions, along with kinetic parameters of their enzymes and the relative fluxes through each reaction (see Code availability). The transformed standard free energy of each reaction is then estimated via the component contribution method [44] at a given pH. Given a catalytic rate constant (${k}_{+}$) and a half saturation constant (${K}_m$,) here assumed to be 200 1/s and 0.2 mM, respectively, the enzyme cost of a reaction is:


$$ E=\frac{1}{k_{+}}\cdot \frac{1+{K}_m/\left[S\right]}{1-\exp \left(\Delta G/ RT\right)} $$

This cost increases when substrate concentration is low because the enzyme is not saturated, and more enzyme is needed to sustain a given flux. Moreover, low substrate concentration and/or high product concentration reduces the free energy dissipated by the reaction, meaning it operates closer to equilibrium and more enzyme is required to facilitate the same net flux. Convex optimization is employed to find the vector of metabolite concentrations that minimizes the total enzyme cost of the pathway (see Code availability).

### Analytical chemistry of culture experiments

**Figure 6:**
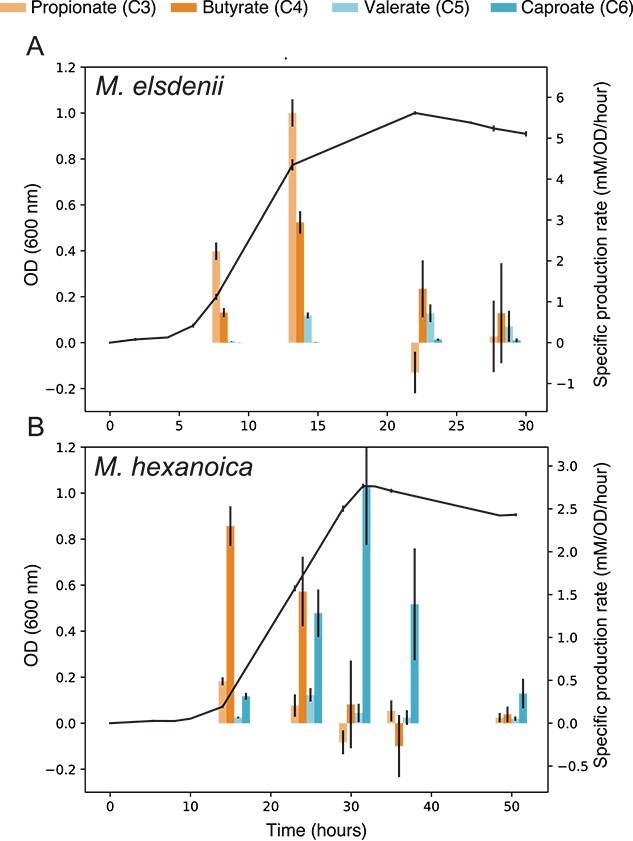
Growth kinetics and specific production rate profiles of *M. elsdenii* (A) and *M. hexanoica* (B) fed with lactate as a sole electron donor. Optical density (OD) measurements are shown with the black line and the left-hand side y-axis. The specific production rates of C3–C6 volatile fatty acids are shown by the colored bars and the right-hand side y-axis. These were calculated by taking the difference in concentration from the previous time point and dividing it by the corresponding time interval and optical density (OD) value. The mean is shown and the error bars represent the standard deviation (n = 3 biological replicates).

Gas chromatography (8900 GC System, Agilent) with tandem mass spectrometry (7000D Triple Quadrupole GC–MS, Agilent) and high-performance liquid chromatography (UltiMate 3000 HPLC system, ThermoFisher) with refractive index detection (RID) were used to generate the data from batch cultures (Figs. 4 and 6). Liquid samples were filtered through a 0.2-micron filter to remove cell debris and stored at −20°C until analysis. The GC–MS was equipped with a DB-FatWax column (Agilent) and used to quantify C2-C8 volatile fatty acids. After thawing samples for GC analysis, they were centrifuged for 5 mins at 10000 RPM, diluted with HPLC-grade water, and formic acid was added to a final concentration of 0.1 M to protonize volatile fatty acids. The run time was 16 mins with the following temperature gradient: 80°C for 1 minute, followed by a 20°C/min ramp until 200°C, 5°C/min until 210°C, and 20°C/min until 250°C and held for 5 mins. The mass spectrometer was operated in dynamic multiple reaction monitoring (dMRM) mode and target analytes were ionized and fragmented by electron ionization. The precursor and product ions of the target analytes were selected using software from Agilent (MassHunter Optimizer). The HPLC was equipped with an Aminex HPX-87H (Bio-Rad) column and used to quantify lactate and glucose, and the column temperature was set to 50°C. The mobile phase was 5 mM sulfuric acid, and the eluent flow rate was set to 0.6 ml/min.

### RUSITEC experiments

The RUSITEC leverages continuous-flow bioreactors inoculated from the rumen that receives solid cattle feed daily [45, 46]. In two replicated experiments, the feed was switched to one with an increased proportion of starch, and the pH in half the reactors was decreased by diluting the buffer by an additional 25% after a 5-day acclimatization phase. Each RUSITEC experiment was carried out using 12 fermenters inoculated with ruminal fluid and solid digesta (trial 1 and trial 2), and the pH was monitored continuously throughout. Further, samples for organic acid measurements and amplicon sequencing were taken over time, whereas metagenomes and metatranscriptomes were collected on experimental day 10. Details on the setup, sampling, measurements (including all additional analytical chemistry methods), sequencing, and analysis of the RUSITEC experiments can all be found in the Supplementary Materials and Methods.

### Statistics and reproducibility

**Figure 7:**
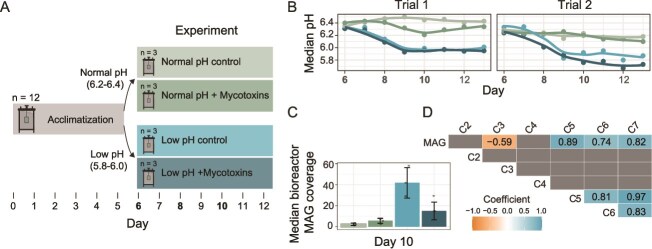
Rumen simulation experiments. (A) The RUSITEC technique was used to enrich two different rumen microbiome states (“Normal pH” and “Low pH”) and test the addition of mycotoxins. The 12 bioreactor vessels were inoculated with rumen fluid and contents and then allowed to acclimatize for 5 days. Buffer was continually flowed into the reactors, and solid feed was added daily to permeable bags that rotated in the reactors. After the acclimatization phase, the feed was changed to represent a high-energy diet, the pH was lowered in half of the reactors by diluting the buffer, and mycotoxins were added to create an additional treatment (n = 3, treatment colors were also used for panels B-D). The bolded days indicate which days were sampled for amplicon sequencing during the experimental phase (Supplementary Fig. S5). (B) The median pH was taken from real-time inline measurements taken throughout the day. Replicated experiments are shown side by side. (C) The median coverage is shown for reads mapped to a high-quality MAG closely related to *M. hexanoica*. The coverage was calculated using metagenomes from experimental day 10 of the second bioreactor experiment. Errors bars show standard deviation (n = 3 metagenomes). (D) The correlation (Pearson) coefficients for significant correlations (*P* value <0.05, t-test on correlation coefficients) between the *M. hexanoica* MAG and fatty acids of different lengths (C2–C7).

The code for reproducing any of the figures and statistical analyses can be found on GitHub (see Code availability). To summarize, we used the Wilcoxon rank-sum test and Benjamini-Hochberg procedure to adjust *P* values for two-group comparisons of the *Megasphaera* species (Fig. 2E, Fig. 3A and C). Before carrying out this test with amplicon sequence data (Fig. 2E and Fig. 3A), the count data were centered log ratio transformed using a pseudocount of 1 to mitigate the effect of compositionality. For the time-course amplicon data analyzed in Fig. 2D, relative abundance values were transformed by taking the square root to improve the visualization of values close to 0. The line shown in Fig. 2D is a generalized additive model as implemented in ggplot2 with basis dimension k = 4 (“stat_smooth(method = “gam”, formula = y ~ s(x, k = 4)”). Further, for the correlations in Figs. 3B and 7D, *P* values were calculated using a t-test on Pearson correlation coefficients. No data was excluded from any of the statistical analyses.

## Results

### Distribution of *Megasphaera* lactate-utilization pathways in the rumen

Several studies have pointed to the importance of lactate utilization in the rumen with a focus on two pathways that lead to either propionate (via the acrylate pathway) or butyrate in the *Megasphaera* [11, 12]. Yet, the role of rBOX (i.e. chain elongation), which shares enzymes with butyrate production and is harbored by the *Megasphaera*, is often overlooked in the rumen and leads to, in addition to butyrate and valerate (C5), the MCFAs caproate (C6), heptanoate (C7), and octanoate (C8). To gain an understanding of how these pathways (acrylate and chain elongation) are distributed across lactate utilizers in the rumen, we first annotated them in a large dataset comprising metagenome-assembled genomes from the rumen in both adult cattle and calves (Fig. 1A and C). This showed that *M. elsdenii* reaches the highest relative abundance among microbes harboring the acrylate pathway occurring in the calf microbiome (Fig. 1B). We also confirmed that the acrylate pathway is conserved across *M. elsdenii* and missing in *M. hexanoica* across our collection of isolate genomes (Supplementary Table S1). The microbes with the potential for lactate-driven chain elongation are also most abundant in the calf, which includes both *M. elsdenii* and *M. hexanoica* (Fig. 1B). This suggests that the *Megasphaera* are important rumen bacteria due to their reasonably unique ability to run the acrylate pathway (leading to propionate) and lactate-driven chain elongation (leading to butyrate, valerate or MCFAs), but also suggests they play much more of a role in the calf than in the adult rumen. Further, the two *Megasphaera* species provide an opportunity to study how the acrylate pathway contributes to niche differentiation, as it is only found in *M. elsdenii*, and the resulting consequences for lactate utilization in the rumen. Before doing so, however, we aimed to gain a general understanding of how the genomic diversity of *M. elsdenii* and *M. hexanoica* is structured and how this structure maps onto a temporal and dietary niche.

### Genotypic structure and *in vivo* dynamics of the rumen *Megasphaera*

We first noticed, based on a concatenated marker gene tree using the available genomes from the two *Megasphaera* species, that there appeared to be somewhat low within-species diversity, consistent with previous claims about *M. elsdenii* [47], even when genomes and metagenome-assembled genomes (MAGs) from different hosts sampled across the world were included (Fig. 2A, Supplementary Table S1). This contrasts with the large amount of differentiation observed between the two species, which can be inferred from phylogenetic branch lengths (Fig. 2A, 35x between vs within species). To quantify the within-species diversity, we calculated the pairwise average nucleotide identity (ANI) between the genomes and MAGs belonging to the same species, which was found to be above 98% on average (Fig. 2B), 3% above the common species threshold of 95% [48]. We confirmed that this was also the case when using high-quality genomes (>90% completeness and < 1% contamination) from the latest release of the genome taxonomy database (v. 226.0, Supplementary Fig. S1A) [49]. Even though the within-species diversity is lower than what we have previously observed in co-existing ruminal *Campylobacter* of the same species (~96.5% ANI) [50], it does not rule out intraspecies units, such as genomovars (typically found at around 99.5% ANI), with distinct habitat distributions [51]. We then applied a model we recently developed and found that the majority of the genome results from clonal descent (Supplementary Fig. S1B) [52]. This suggested that genome-wide sweeps, which occur when an adaptation leads to a single genome outcompeting others within its niche, have structured the *Megasphaera* genotypically (Supplementary Fig. S1C). To test this within a single habitat (i.e. single host and geographical location), we compared the strain-level diversity present in the set of metagenomes from calves from the same herd (Fig. 2C). This showed that diversity has been purged across the genome with scattered, local increases in the density of SNPs, which is consistent with genome-wide selective sweeps (Supplementary Fig. S1D) [50, 52, 53]. Also, the nucleotide diversity that has accumulated indicates strong purifying selection in both species (Supplementary Fig. S1E and F). Taken together, our analysis suggests that diversity within *Megasphaera* species has been maintained low by genome-wide sweeps, which is likely the result of strong selection [54], and that the two species have been stably co-existing across multiple mammalian hosts. This does not rule out host-specific adaptations at the population level, which have been observed in other rumen bacteria [55], but it implies that the two highly differentiated species are optimized to distinct, stable niches. We, therefore, first hypothesized that the two *Megasphaera* species are differentially adapted to distinct stages during the growth and development of the host animal.

One convenient aspect of comparing two highly differentiated species is that the 16S rRNA gene sequence can be used to monitor their dynamics over time. We, therefore, compiled amplicon data from multiple studies to monitor the abundance of *M. elsdenii* and *M. hexanoica* in the cattle rumen (Fig. 2D). We further included the data from the swine gut to assess whether the dynamics are specific to the rumen or observed in other mammalian hosts. Both *Megasphaera* species appear to peak in abundance during early animal development (before reaching 100 days in age) in both the cattle rumen and swine gut (Fig. 2D). This pattern is also observed for the *Megasphaera* in a recent study that identified core successional species (operational taxonomic units) that cluster in an age-dependent manner (Supplementary Fig. S2A) [22]. Further, even though *M. elsdenii* is generally more abundant than *M. hexanoica*, both are enriched in the adult rumen by a high-starch diet, which is known to select for lactic acid bacteria (Fig. 2E) [56, 57]. These observations ultimately reject the hypothesis that the two *Megasphaera* species are adapted to different stages of host development and show that they share a certain degree of ecological overlap. We therefore asked if their distinct niches involve specialization to key resources. As the representative strains from both *Megasphaera* species are known to utilize lactate to different degrees [9, 29, 58, 59], we tested for differential association with rumen states that involve increased lactate production and utilization.

### Differential associations and gene expression in the rumen *Megasphaera*

It is well established that rumen microbiomes generating less methane are enriched for organisms involved in lactate production and utilization, including the *Megasphaera* [11–13]. Therefore, we first compared *in vivo* associations of the two species in multiple studies and found only *M. hexanoica* to be more consistently associated with a low-methane, high-lactate state in the adult rumen relative to *M. elsdenii* (Fig. 3A). However, this observation conflicts with *in vitro* data, as *M. elsdenii* preferentially consumes lactate over glucose and grows rapidly when doing so^28^. Indeed, the culture media from these *in vitro* experiments contain high millimolar (mM) substrate concentrations, whereas lactate is commonly found in the micromolar (μM) range in the adult rumen under normal (non-acidotic) conditions [15]. The calf rumen, in contrast, does often reach mM lactate concentrations [60], which led us to correlate the two *Megasphaera* species with higher lactate concentrations in the calf (Fig. 3B). Again, this showed a stronger association with lactate for *M. hexanoica* compared to *M. elsdenii*. We, therefore, considered that the *in vitro* substrate preference of *M. elsdenii* for lactate may not reflect preferences in the rumen and hypothesized that *M. hexanoica* primarily utilizes lactate, whereas *M. elsdenii* primarily utilizes sugars *in vivo*.

We began testing the hypothesis that *M. hexanoica* and *M. elsdenii* have distinct substrate preferences by comparing the *in vivo* transcription of marker genes of glycolysis and lactate utilization in the calf rumen. We used *gapA* for glycolysis, a central enzyme in the main glycolytic pathways, and *larA*, a lactate racemase that has been shown to be upregulated during lactate utilization *in vitro* by *M. elsdenii* [29]. Here, we saw that *M. elsdenii* and *M. hexanoica* preferentially express the genes for sugar and lactate utilization, respectively, *in vivo* (Fig. 3C). Among other highly expressed genes by both *M. elsdenii* and *M. hexanoica* in the calf rumen were marker genes for the rBOX pathway (i.e. chain elongation), whereas the acrylate pathway showed no evidence of transcriptional activity (Fig. 3C, Supplementary Fig. S2B). Based on these data and previous results with *M. elsdenii* [28], we hypothesized that chain elongation, which results in butyrate and MCFA production, is more active in *M. elsdenii* when utilizing glucose. In contrast, chain elongation is more active in *M. hexanoica* when utilizing lactate. To test the hypotheses, we assessed growth in the presence of glucose and lactate.

### *Megasphaera* fermentation patterns in the presence of both glucose and lactate

We compared the two *Megasphaera* species in media containing both glucose and lactate for two reasons: (i) to confirm previous results with *M. elsdenii* [28] and (ii) to evaluate whether *in vitro* substrate preferences and end product profiles throughout the growth curve support the hypothesis that chain elongation is more active in *M. elsdenii* and *M. hexanoica* during glucose and lactate utilization, respectively. Substrate utilization and product formation were consistent with previous *in vitro* results with *M. elsdenii*, as lactate was consumed rapidly before glucose during early exponential growth (Fig. 4A, top panel) and coincided with the production of propionate via the acrylate pathway (Fig. 4A, bottom panel) [28]. Later in the growth curve, when lactate is depleted and *M. elsdenii* primarily consumes glucose (Fig. 4A, top panel), we observe butyrate, valerate (C5), and caproate (C6) production, which are the result of chain elongation (Fig. 4A, bottom panel). This pattern has been observed for multiple strains of *M. elsdenii* [61], and again suggests that the acrylate pathway, which was not observed to be expressed in the calf rumen (Supplementary Fig. S2B), is favored by the initial *in vitro* culture media conditions. The data also further support the hypothesis that chain elongation is more active during glucose consumption in *M. elsdenii*. For *M. hexanoica*, no consumption of glucose was observed, and lactate was utilized later and more slowly, which we observed in multiple strains (Fig. 4B, top panel, Supplementary Fig. S3A). This coincided with chain elongation and the production of butyrate and more extended MCFAs, including caproate (C6) and octanoate (C8) (Fig. 4B, bottom panel). Consistent with the lack of glucose consumption, it has been reported that *M. hexanoica* grows poorly on glucose [35]. Thus, *in vitro,* in the presence of lactate and glucose, *M. elsdenii* preferentially utilizes lactate via the acrylate pathway but switches to chain elongation at some point during lactate depletion and glucose consumption. This latter metabolism (i.e. chain elongation paired with glycolysis) is what appears to be primarily run *in vivo* in the calf rumen, based on the above transcriptomic results, but this does not exclude a degree of co-utilization of lactate and glucose (Fig. 3C). In contrast, *M. hexanoica* seems to rely on lactate-driven chain elongation *in vitro* (in the presence of glucose) and *in vivo* (Fig. 3C, Fig. 4B).

Our goal then was to better understand how the different lactate utilization strategies are related to the ecology of the two species. In line with our previous work [37], we hypothesized that the acrylate pathway enables a high-rate, low-energy yield growth strategy during lactate accumulation. To investigate this hypothesis, we first modeled the proteomic cost of the acrylate and chain elongation pathways.

### Predicted growth strategies based on proteome allocation

Using a model that relates thermodynamics and proteome allocation [43], we aimed to compare the enzyme cost per ATP flux between the acrylate pathway and chain elongation. Indeed, during the catabolism of key carbon sources, such as lactate, there is often an inverse relationship between ATP yield and proteomic cost per unit of ATP flux of the enzymes involved in the catabolic pathways [43]. A pathway that conserves more energy tends to have enzymatic steps that operate close to equilibrium, as the available free energy serves to both drive these reaction steps forward and synthesize ATP. Because the ratio of forward to reverse flux through a thermodynamically constrained reaction is smaller (compared to a reaction operating far from equilibrium), a larger portion of the enzyme pool catalyzing a constrained reaction is “wasted” in facilitating reverse flux. This means that more enzyme is required to carry net forward flux through a pathway that conserves energy more efficiently [43]. Resource allocation theory then predicts that more enzyme investment into catabolism necessitates less investment elsewhere, such as in anabolism [62]. Therefore, higher energy yield strategies often cause slower growth [37]. Using Enzyme Cost Minimization (ECM), we predict a substantial increase in enzyme cost per ATP flux with the production of longer carbon chain fatty acids (from propionate to butyrate through to MCFAs) from lactate (Fig. 5). For example, the enzyme cost per ATP flux produced via rBOX is 4–7 fold higher than that compared to the acrylate pathway (Fig. 5). Conversely, the acrylate pathway yields 0.33 mole ATP per mole lactate, whereas rBOX yields 0.5. The main consequence of this analysis is that, because *M. hexanoica* does not harbor the enzymatically “cheaper” acrylate pathway, *M. elsdenii* should have a higher maximum possible growth rate on lactate at the expense of ATP yield. Consistent with this expected higher growth rate, *M. elsdenii* harbors an extra copy of the 16S rRNA operon and has a ~ 1.5-fold higher inferred microbial population replication rate (Supplementary Table S2) [63–65]. Also, as we have shown previously, the ECM analysis predicts that the growth rate on lactate should be inversely related to the extent of chain elongation [37]. To test whether the different pathways correspond to the predicted growth strategies favoring rate or yield, we evaluated flux through the acrylate and rBOX pathways when grown with lactate as the main electron donor for both *Megasphaera* species.

### *Megasphaera* growth rates on lactate and their relation to pathway flux

We found that SCFA and MCFA production throughout growth on lactate as the primary electron donor is largely consistent with predictions based on ECM, suggesting that *M. elsdenii* is overall adapted to a higher-growth rate strategy on lactate with respect to *M. hexanoica*. The flux through the acrylate and rBOX pathways producing MCFAs of different lengths shifts throughout the growth curve (Fig. 6). Shorter-chain fatty acids are produced rapidly during the early exponential phase, but their production slows as growth slows (Fig. 6). In contrast, with the slowing of growth, the rate of production of longer-chain fatty acids increases in order of chain length. This entire pattern is shifted to the shorter-chain fatty acids for *M. elsdenii*, which predominantly produces propionate during the early exponential phase and switches to mainly butyrate, along with small amounts of longer-chain fatty acids (C5 and C6) by the end of the growth curve (Fig. 6A). *M. hexanoica*, in contrast, predominantly produces butyrate during the early exponential phase and then later switches to predominantly caproate (C6, Fig. 6B). In this experiment, *M. hexanoica*, despite lacking the acrylate pathway, did produce small amounts of propionate, which may stem from amino acid catabolism. Nevertheless, *M. elsdenii* maximizes the growth rate on lactate by running the acrylate pathway and less extensive chain elongation relative to *M. hexanoica*. The question then remains as to when the higher yield strategy on lactate (i.e. lactate-driven chain elongation) outcompetes the higher rate strategy (i.e. the acrylate pathway) when the two *Megasphaera* species compete for lactate. In this context, the growth rates of the two species over a wide range of lactate concentrations suggest that *M. hexanoica* may have a higher affinity for lactate (Supplementary Fig. S3B). Considering this potentially increased affinity [52, 54], we would expect the higher yield strategy to be more competitive during low lactate fluxes where lactate is not observed to accumulate and, thus, supports relatively slow growth rates [62, 66]. Another aspect may be the effect of pH, which when low, could further increase the competitiveness of lactate-driven elongation.

### Interaction of lactate utilization pathways in the *Megasphaera* with pH

The interaction of the lactate utilization pathways in *Megasphaera* with pH may be important for understanding when one of the pathways is favoured. Indeed, one of the key differences between the acrylate pathway and lactate-driven chain elongation is the balance of protons in the overall reactions. Specifically, the acrylate pathway is balanced in terms of protons, whereas chain elongation consumes protons. Consistent with this, *M. hexanoica* showed an increased ability to raise the pH compared to *M. elsdenii*  *in vitro* (Supplementary Fig. S4). In fact, *M. elsdenii* decreased the media pH overall when both glucose and lactate were present (Supplementary Fig. S4A). When grown on lactate as the main carbon source with a starting media pH of 5.5, both species increased the media pH, but the effect was larger for *M. hexanoica* (Supplementary Fig. S4B). Consistent with this, it has been shown that a pH below 6 progressively selects for chain elongation, which supports a basic thermodynamic analysis [32]. As the pH of the calf rumen is consistently found to be below 6 [18, 19, 60], decreased pH may increase the competitiveness of *M. hexanoica* on lactate in the calf as it thermodynamically favors chain elongation.

### Enrichment of *M. hexanoica* in simulated rumen microbiomes with depressed pH

In parallel with studying differentiation in lactate utilization pathways in the *Megasphaera*, we conducted long-term *in vitro* experiments—also known as the rumen simulation technique (RUSITEC) [ 45, 46, 67]. The main goal of this experiment was to study the microbiome during pH depression, which is expected to enrich for chain elongators [32], but a second treatment included the addition of a common ruminant feed contaminant, i.e. mycotoxins. This is because many mycotoxins remain stable in the rumen during pH depression and are thought to negatively impact the rumen microbiome [68–70]. In short, the RUSITEC experiments showed that pH depression can enrich *M. hexanoica* from rumen fluid without the accumulation of lactate, but prevalent mycotoxins inhibit its growth (Fig. 7). Specifically, during the experimental phase of the RUSITEC experiments (after the acclimatization phase), the pH of reactors was consistently lowered by ~0.4 in both experimental repetitions by diluting the inflowing buffer, reaching a lower pH of ~5.8–6 (Fig. 7A and B). Based on amplicon sequence variants (ASVs), decreasing the pH of the reactors highly enriched for an ASV classified as *M. hexanoica*, which was also negatively impacted by the mycotoxins (Supplementary Fig. S5). Additionally, the dominant mycotoxins in the reactors were more stable at low pH, and we confirmed the inhibitory effect of two mycotoxins (deoxynivalenol and aurofusarin) on *M. hexanoica*  *in vitro* (Supplementary Figs. S6 and S7) [68]. We thus suggest that mycotoxins should be considered as potential inhibitors of *M. hexanoica*, especially in the calf rumen where pH is low and thus mycotoxins are more stable.

In the RUSITEC experiments, there was also a reproducible trend observed in the low pH reactors in which propionate decreased, and valerate (C5) increased (Supplementary Fig. S8A). As this pointed to chain elongation by *M. hexanoica* (utilizing propionate as a substrate to produce odd-chain MCFAs), we further re-measured valerate (C5), and measured caproate (C6) and heptanoate (C7), in addition to quantifying *M. hexanoica* using metagenomics on day 10 of the experiment (Fig. 7C, Supplementary Fig. S8B). This recovered a single metagenome-assembled genome (MAG) from the individual bioreactors representing *M. hexanoica* (Supplementary Table S3, Supplemental Table S4)*.* The coverage of the *M. hexanoica* MAG was consistent with the amplicon sequencing data and correlated significantly with the products of chain elongation in the rumen (Fig. 7C and D, Supplementary Fig. S5). Together, the enrichment of *M. hexanoica* in the RUSITEC experiments with decreased pH is consistent with previous results that enrich for chain elongators [32, 71].

The RUSITEC experimental data also appear to support the hypothesis that *M. hexanoica* is adapted to low lactate availability. To start, some of the other most abundant bacteria in the low pH reactors were several lactic acid bacteria (LAB, ex. *Bifidobacterium* and *Lactobacillus*, Supplementary Table S5). This implied that the low pH reactors may be undergoing lactate cross-feeding. Supporting this, we sequenced transcriptomes from the reactors and observed the lactate racemase in *M. hexanoica* to be one of the top expressed genes (Supplementary Fig. S9), which is consistent with lactate utilization [29]. We were also unable to detect lactate by GC–MS at day 10 in the reactors. Along with the correlation between *M. hexanoica* and chain elongation products (Fig. 7C), this suggests that *M. hexanoica* can come to dominate when running lactate-driven chain elongation during co-enrichment with LAB and without lactate accumulation. This, along with the expected ATP yields of the different pathways, corroborates the idea that *M. hexanoica* has adapted to low lactate fluxes and that its growth strategy, as it relies on more extensive chain elongation, is favored by a depressed pH (<6) in the rumen. In contrast, *M. elsdenii* can obtain a clear growth rate advantage when lactate accumulates by running the acrylate pathway. Indeed, based on both our above modeling and the *in vitro* and *in vivo* data, we suggest that when lactate is scarce, the higher yield but enzymatically costly strategy of chain elongation to produce butyrate and MCFAs may be preferable to the lower yield, enzymatically cheaper strategy of propionate production.

## Discussion

Since its initial isolation, *M. elsdenii* has been studied in the context of utilizing lactate to produce SCFAs and prevent acidosis [72]. Based on this, probiotic products containing *M. elsdenii* are on the market, but their effectiveness in improving rumen microbiome function is debatable [72, 73], perhaps due to a lack of understanding of its unique niche. More convincingly, *M. elsdenii* has been associated with ruminant feed efficiency, where more carbon enters the animal as SCFA rather than being expelled as methane [11–13]. While the extent to which *M. elsdenii* contributes overall has been questioned due to its low relative abundances, the role of lactate metabolism in efficient rumen microbiomes is strongly supported [11–13]. In terms of end products, valerate and caproate often correlate with high feed efficiency in these studies, and are associated with a subset of heritable core rumen microbes [74], which points to the potential importance of chain elongation. Further, valerate, like propionate, contributes to gluconeogenesis in the host and is highly predictive of a rumen state at the risk of acidosis [75, 76]. Therefore, more attention should be given to chain elongators in the rumen, especially those that are abundant in adult animals.

Lactate has been shown to accumulate to higher concentrations in the calf than in the adult rumen, and it is in the calf that both *Megasphaera* are simultaneously enriched. The *Megasphaera* are also enriched in the adult rumen during states of increased lactate production and utilization, but at much lower relative abundances. We show that this enrichment can often be attributed to an increase in *M. hexanoica* rather than *M. elsdenii*, as *M. hexanoica* was more significantly enriched in rumen lactate production/utilization states. Similarly, *M. hexanoica* appears to have a higher affinity for lactate and was enriched in RUSITEC bioreactor experiments, where no lactate accumulation was observed. These results suggest that when lactate is scarce, the higher yield but proteomically costly strategy of chain elongation may outcompete the lower yield, proteomically cheaper strategy of propionate production via the acrylate pathway. It may be then that *M. hexanoica* thrives when lactate is scarce in the rumen by leveraging a higher yield catabolic strategy, i.e. chain elongation. This niche dimension may be important, as we observed that the genes for lactate utilization and rBOX are more highly expressed than those for glycolysis. In terms of the acrylate pathway harbored by *M. elsdenii*, we did not observe expression *in vivo*, but there is some evidence that the pathway is expressed at low levels in the adult rumen, although less so than butyrate production, and with non-stringent read mapping [11]. It would be interesting to know if acrylate pathway expression in the adult rumen is due to highly transient or local accumulation of lactate. Indeed, there is evidence in adult cows entering lactation that lactate concentrations are highly variable, spiking up to 15 mM, throughout the day [77]. There are also relevant trends in the bioprocess literature where *M. hexanoica* is often used in continuous flow reactor experiments [78], while *M. elsdenii* is used in a batch reactor bioprocess where lactate accumulates, which appears consistent with their distinct niches [79]. We therefore suggest that *M. elsdenii’s* unique niche involves growing rapidly during transient fluctuations in lactate availability, which is distinct from *M. hexanoica’s*, which lacks the acrylate pathway. Also, we discuss these general differences in lactate utilization via the acrylate pathway and lactate-driven chain elongation at the species level, as these physiological traits seem to be conserved in multiple strains from different sources [28, 29, 35, 58, 61, 72, 80–82]. It remains likely, however, that finer-scale units exist, especially across habitats, and are differentiated in various specific traits, which could include aspects of lactate utilization and other key resources, such as sugars (see Supplementary discussion).

In addition to differential adaptation to resource fluctuations driven by trade-offs, a mechanism possibly contributing to co-existence and niche differentiation is that *M. hexanoica* may form cross-feeding relationships with certain lactate-producing microbes with similarly constrained growth rates. This is, to a degree, consistent with the stress gradient hypothesis [83], whereby ecological interactions are expected to be more positive as stress, such as resource limitation, increases. But it remains an open question to what extent *M. elsdenii* and *M. hexanoica* are adapted to cross-feeding with other microbes [84]. It does seem likely that adaptations in lactate producers, many of which involve pH tolerance, could lead to the establishment of cross-feeding networks [85]. In fact, the rate-yield trade-off in the *Megasphaera* is reminiscent of Hungate’s observation that *Streptococcus bovis*, a lactic acid bacterium from the rumen, switches to mixed acid fermentation at low growth rates [86]. Also, it would seem that slow-growing *M. hexanoica* requires some sort of physical association with feed particles, such as dietary starch, as these have a slower passage rate than the liquid passage rate in the calf, which is extremely fast (~40–46% of ruminal fluid per hour) [87, 88]. In line with this, a recent experiment showed that restricting solid feed to calves leads to a decline in the *Megasphaera* [89].

We propose that the *Megasphaera* play an outsized role in the calf rather than the adult rumen, as the *Megasphaera* are among the dominant lactate utilizers in the calf. In addition to potentially exerting priority effects on rumen development [22], the *Megasphaera* may impact acidosis in the calf rumen, which is often provided with a high-grain diet and maintains a low pH through weaning [21]. Indeed, even a small decrease in pH in the rumen can lead to a considerable decrease in daily weight gain and several health issues, such as hyperkeratosis and ulcers [21]. Although both *Megasphaera* can contribute to preventing pH depression, the chain elongation pathway, unlike the acrylate pathways, leads to proton consumption and thus has more potential to raise the pH and generally consume reductant. Thus, the inhibition of lactate-driven chain elongation, for which *M. hexanoica* is more specialized, should be avoided. Here, several prevalent mycotoxins pose a particular risk as they are common contaminants in grain-based diets, poorly degraded at pH values commonly found in the calf rumen, and inhibit *M. hexanoica.* To selectively enrich for *M. hexanoica*, mechanisms contributing to MCFA resistance could be leveraged, as it was initially isolated on selective media containing high levels of caproate [82]. In the end, by showing how *M. elsdenii* and *M. hexanoica* partition lactate, a key resource in the rumen, we provide an example of how comparing related, co-existing microbial populations with distinct niches can provide insight for modulating rumen microbiome function.

## Supplementary Material

Strachan_et_al_ISME_supp_information_reviewed_final_no_line_nums_with_figs_wraf147

Supplementary_Table_1_final_wraf147

Supplementary_Table_2_final_wraf147

Supplementary_Table_3_final_wraf147

Supplementary_Table_4_final_wraf147

Supplementary_Table_5_final_wraf147

Supplementary_Table_6_final_wraf147

## Data Availability

The amplicon and metagenomic sequencing data from the bioreactor experiments are available on NCBI under the BioProject ID PRJNA1274365.

## Code availability

Scripts to regenerate all figures can be found at https://github.com/cameronstrachan/RumenMegasphaera2025 (Permanent DOI: 10.5281/zenodo.15629839). The repository further includes commands for reproducing bioinformatic analysis and files with selected reference sequences.
