# Supplementary material for: Distinct lactate utilization strategies drive niche differentiation between two co-existing Megasphaera species in the rumen microbiome
Source: ISME J. 2025 Jul 14;19(1):wraf147. doi: 10.1093/ismejo/wraf147 (PMC12342382; doi:10.1093/ismejo/wraf147)
Supplement: Strachan_et_al_ISME_supp_information_reviewed_final_no_line_nums_with_figs_wraf147 [file strachan_et_al_isme_supp_information_reviewed_final_no_line_nums_with_figs_wraf147.pdf]

Supplementary Information for "Distinct lactate utilization strategies drive niche differentiation between two co-existing *Megasphaera* species in the rumen microbiome".

Cameron R. Strachan, Connor M. Bowers, Byung-Chul Kim, Tea Movsesijan, Viktoria Neubauer, Anna J. Mueller, Xiaoqian A. Yu, Fátima C. Pereira, Veronika Nagl, Johannes Faas, Martin Wagner, Qendrim Zebeli, Paul J. Weimer, Pieter Candry, Martin F. Polz, Christopher E. Lawson, Evelyne Selberherr

Contents

Supplementary Materials and Methods
Supplementary Discussion
Supplementary References
Supplementary Figures S1 – S9
Legends for Supplementary Tables S1 – S6

Supplementary Materials and Methods

Analysis of publicly available genomes and metagenomes

To assess the distribution of lactate utilization pathways, as shown in Figure 1, a database of MAGs using metagenomes from calf and adult rumen was compiled. Metagenomes collected from the calves by Malmuthuge *et al.* [3] were first assembled using SPAdes (v. 3.15.2)[4] and mapped using BWA-MEM (v. 0.7.17)[5]. MAGs were subsequently generated using Metabat2 (v. 2.12.2)[6] and added to those that were generated by Stewart *et al.* [7], who used metagenomes from the adult rumen. Genomes from known lactate chain elongators, including *M. elsdenii*, *M. hexanoica*, *Pseudoramibacter alactolyticus* and *Ruminococcaceae* CPB6 were then combined with MAGs as internal controls. The resulting database of MAGs and genomes was first dereplicated at 98% ANI using fastANI (v. 1.33)[8] where the most complete genome as per checkM (v. 1.1.3)[9] was selected as representative. After replicating the database, the MAGs and genomes were classified using the classified workflow from the genome taxonomy database toolkit (GTDB-Tk, v. 2.1.0)[9], which was also used to generate a concatenated marker alignment. A phylogenetic tree was then built using the LG model in iqTree (v.1.6.12, flags; "-m LG+F -bb 1000 -alrt 1000") with ultrafast bootstraping[10].

To screen for the metabolic potential to run the acrylate pathway and lactate-driven chain elongation, we compiled a set of marker proteins from the UniProt database (acylate pathway, butyrate production, reverse beta-oxidation, and lactate racemization; the markers are available as a fasta file, see Code Availability). We then checked whether a homolog for each marker gene for the pathways was present for each of the following pathways in the dereplicated MAG and genome database. This was done by aligning open reading frames from the database, predicted with prodigal (v.2.6.3), to the marker proteins using blastp (v.2.5.0+) with a 95% alignment length and 40% amino acid identity cutoff. Additionally, the overall potential for lactate-driven chain elongation required the presence of an annotated lactate racemase. The approach recovered all known lactate chain elongator genomes included. All candidate chain elongator MAGs and genomes were then quantified by mapping metagenomes from Stewart *et al.*[7] (adult rumen,

n=21) and Malmuthuge *et al.*[3] (calf rumen, n=18) using BWA-MEM (v. 0.7.17)[5]. Mapped reads were then blasted against the predicted genes using blastn (v.2.5.0+) and were annotated using EggNOG (v.2.1.12, database v.5.0.2, Diamond v.2.1.8)[11]. Reads that aligned to annotated ribosomal proteins with alignment identity over 70% and nucleotide identity over 95% were counted. The counted reads were finally normalized to the total gene length of the ribosomal proteins and total reads per metagenome.

MAGs and genomes belonging to the *Megasphaeraceae* were identified using the GTDB-Tk (v. 2.1.0), which also generated the alignment underlying the concatenated marker tree in Figure 2A. The phylogenetic tree was then built using the LG model in iqTree (v.1.6.12, flags; "-m LG+F -bb 1000 -alrt 1000") with ultrafast bootstraping[10]. Next, the clonal fraction was calculated using a recently developed mixture model of pairwise SNP distributions[12]. The average nucleotide identity between the genomes and MAGs in Figure 2B and Supplementary Figure 1A was calculated using fastANI. In Supplementary Figure 1A , all genomes from GDTB[1] R226 belonging to either *Megasphaera elsdenii* or *Caecibacter massiliensis* with more than 90% completeness and less than 1% contamination (as per checkM[9]) were downloaded from the NCBI assembly database. To analyze population-level diversity (within a single host, Figure 2C, and Supplementary Figure 1D, E and F) we used inStrain (v1.9.0, flags: "--pairing_filter non_discordant"). Four metagenomes from the calf were used for the inStrain analysis (Malmuthuge *et al.*[3]), with reference genomes for both *M. hexanoica* and *M.elsdenii* (strains MH and T81, respectively), and compared using the *compare* function with default settings. The same metagenomes were also used to calculate the inferred microbial population replication rates iRep and bPTR (provided in Supplementary Table 2) using the corresponding Python scripts provided by the developers of iRep[13].

Analysis of publicly available amplicon and transcriptomic data

Amplicon data was compiled and analyzed (Figure 2D and E, and Figure 3A and B) from O'Hara et al.[14], Kodithuwakku et al.[15], Gaire et al.[16], Wang et al.[17], McGovern et al.[18], Kamke et al.[19], Stepanchenko et al.[20] and Dill-McFarland et al.[21]. The data were processed using the qiime2 environment (v. 2021.4.0)[22]. The forward reads from all datasets were denoised using dada2 (see

Code Availability for specific trimming parameters) and blasted (blastn, v.2.5.0+) against *M. hexanoica* and *M. elsdenii* reference genomes (strains MH and T81, respectively) to identify amplicons assigned to each species (>99.5% identity).

The data presented in Figure 3C and Supplementary Figure 2B are based on the analysis of metatranscriptomes published by Park et al.[23]. The forward reads were competitively mapped to the same reference genomes using BWA-MEM (v. 0.7.17)[5]. Open reading frames (ORFs) were then predicted and annotated using prokka (v. 1.14.6)[24] and reads mapping to predicted ORFs (as above) were counted using htseq-count (v. 0.11.3)[25].

Time series of batch cultures with lactate and glucose

Following colony picking on agar plates, *Megasphaera elsdenii* (DSM20460) and *Megasphaera hexanoica* (DSM106893) were grown anaerobically in serum bottles with 100 mL modified ATCC 2107 lite media containing 50 mM sodium DL-lactate, 50 mM sodium acetate and 50 mM glucose, with an initial pH of 6.0. Both organisms were tested in quadruplicate vials, along with triplicates of cultures without lactate or glucose. Intermittent sampling of 1 mL was performed using syringes to measure optical density at 600 nm, C2-C8 fatty acid concentrations via Gas Chromatography with Mass Spectrometry (GC/MS, Supplementary Materials and Methods), and lactate and glucose concentrations via High-Performance Liquid Chromatography (HPLC, see analytical chemistry section below).

Time series of batch cultures with lactate and fructose using M. hexanoica MH

An additional batch culture experiment was conducted (Supplemental Figure 3A), which used a different strain of *M. hexanoica*. *M. hexanoica* MH was ordered from the Japan Collection of Microorganisms with reference number JCM 31403. Following colony picking on agar plates, both strains (DSM20460 and MH) were grown in liquid media using tryptic acid broth supplemented with L-cystein as a reducing agent. Specifically, the base liquid media contained 2.1 g/L of D-fructose, 10 g/L tryptone, 6 g/L soy peptone, 2.5 g/L potassium phosphate, and 1 g/L L-cysteine, with an initial pH of 6.0. To test the effect of lactate, 0.6% v/v sodium lactate was added to the base media and the experiment was conducted at 37°C without shaking. Hungate

tubes were filled with 14 mL of media, inoculated 1:100 from overnight starter cultures and sampled them with a syringe and needle for OD and lactate measurements. The OD610 measurements of 100 µL of media were conducted using a Synergy H1 plate reader in a clear, flat-bottom 96-well plate. For the L-lactate measurements, the BioVision Lactate Colorimetric/Fluorometric Assay was used as per the manufacturer's instructions.

Time series of batch cultures grown on lactate

Following colony picking on agar plates, *Megasphaera elsdenii* (DSM20460) and *Megasphaera hexanoica* (DSM106893) were grown anaerobically in Hungate tubes with 10 mL modified ATCC 2107 media containing 5 g/L sodium DL-lactate and 3 g/L sodium acetate. For both organisms, three different initial pHs were tested (5.5, 6.0, 6.5) in triplicate, along with duplicates of uninoculated tubes and cultures without lactate. Cultures were incubated at 37 °C. Optical density at 600 nm was measured, and 300 uL samples were taken with syringes in an anaerobic chamber (Coy Labs, USA) intermittently (intervals of 3 to 8 hours) until growth halted. C2-C8 fatty acid concentrations were measured via GC/MS, and lactate concentration was measured via HPLC (see analytical chemistry section below).

Characterization of growth kinetics at different lactate concentrations

A protocol for high-throughput kinetic characterization of microbial growth, which was recently applied to *Clostridium kluyveri,* was adapted here[26]. Using a liquid handling robot in an anaerobic chamber, 200 uL suspension cultures of *Megasphaera elsdenii* (DSM20460) and *Megasphaera hexanoica* (DSM106893) were prepared with 18 different lactate concentrations ranging from 0 mM to 150 mM in 96-well microplates. ATCC 2107 lite with 3 g/L sodium acetate was used as the base medium. Each lactate concentration had four corresponding replicates per plate. Three plates were prepared per organism, each being inoculated with a separate isolate obtained from streak plating. Plates were incubated anaerobically in an automated incubator, which transferred plates to a spectrophotometer every 1 to 2 hours for the measurement of each well's optical density at 600 nm. This allowed for growth curves to be made for each well. All OD readings were normalized to the reading of each curve, and the

logarithm of this quotient was taken. Instantaneous specific growth rates were approximated by taking the derivative via forward difference:

$$\mu(t) = \frac{d}{dt}\ln\left(\frac{OD(t)}{OD_{min}}\right) = \frac{1}{OD}\frac{dOD}{dt}$$

The maximum of this derivative is the maximum specific growth rate of the curve. Through manual curation, it was ensured that an initial phase of background growth, which was also observed in wells without supplemented lactate, was excluded when evaluating the maximum growth rate. Maximum specific growth rates were averaged across all replicates.

RUSITEC experiments

The twelve reactors used in the RUSITEC experiments were inoculated with ruminal fluid and solid digesta, which were obtained from nonlactating rumen-cannulated Holstein cows kept at the Dairy Research Station of the University of Veterinary Medicine Vienna. Their usage did not require ethical approval. Donor cows were fed with hay and grass silage and were kept according to Austrian guidelines for animal welfare (Federal Ministry of Health, Austria, 2004, BGBl. II Nr. 485/2004). Each reactor then received a mesh bag of 20 g of feed daily, the flow rate of the buffer was set as to achieve a 70% daily turnover and 23 mL of a solution containing calcium and magnesium was added each day (Supplementary Table 6) [27]. All components of the feed except the alfalfa pellets were mixed for 1 hour before being combined with the alfalfa pellets in the mesh bags (Supplementary Table 6). The experiment consisted of an acclimatization period of five days, during which all reactors received a high-fiber diet (Supplementary Table 6) followed by an experimental period of eight days. At the beginning of the experimental period, the feed was switched from ‘low starch’ to ‘high starch’ (Figure 7A, Supplementary Table 6). Simultaneously, four treatment groups (n=3) were created. This was done by first adding 0.33 g of a mycotoxin containing fungal extract to simulate a realistic *in vivo* situation of approximately 5 ppm deoxynivalenol (DON, extract also contained zearalenone (ZEN), culmorin (CUL) and aurofusarin (AURO) at lesser amount, see next section). Secondly, the inflowed buffer was diluted (75%) to imitate pH depression during SARA. Thus the 4 treatment groups included reactors with/without mycotoxins and pH depression. Liquid samples were taken daily and stored at -20°C for DNA sequencing and chemical analysis. For organic acid measurements, the

samples were further centrifuged (16,600 x g for 30 min), and the supernatant was removed before freezing. The organic acids were measured by HPLC and GC-MS, while mycotoxins and associated metabolites were measured by LC-MS/MS (see analytical chemistry section below). Further, samples for RNA sequencing were taken on experimental day 10, which were flash-frozen in liquid nitrogen and stored at -80°C.

Preparation of Mycotoxin containing extract

To prepare the mycotoxin containing fungal extract, corn kernels (200 g, feed grade) were mixed with 200 mL deionized water and soaked for 60 minutes at room temperature (23°C ±1°C) in polypropylene plastic bags (200 x 300 mm, Roth) before autoclaving (121°C for 20 min). The autoclaved corn material was then transferred sterilely into disposable PD 1200 Microboxes (Nevele). *Fusarium graminearum* was inoculated using 100 µL of a conidial suspension (1.0 x 10^5 conidia/mL) per box, followed by cultivation at 25°C for 4 weeks in the dark. The cultures were then exposed to 75°C for 2 hours (while the boxes were still closed) and subsequently to 70°C (with the boxes open) for the next 4 days. The dried cultures were then transferred into a 30 L bag, thoroughly mixed and manually crushed. The material was then ground in a grinder (Retsch GM 200) at 10 000 rpm for 10 seconds, sieved and further homogenized before final sampling. A mixed sample (10 g) was then transferred into 250 mL capacity Erlenmeyer flasks and extracted with 100 mL of solvent (ACN:H2O:AcOH, 70:29:1) by rotary agitation at 160 rpm for 60 min. An aliquot of 100 µL was taken, diluted, and filtered before LC-MS analysis. The extract was found to contain, as the major mycotoxins detected, zearalenone (ZEN), deoxynivalenol (DON), culmorin (CUL) and aurofusarin (AURO) at concentrations of 609.7, 1579.1, 229.1 and 223.9 ppm, respectively. The 0.33 g of the extract was added to the reactor to achieve approximately 5 ppm DON.

DNA and RNA extraction from RUSITEC samples

The DNA samples taken from three time-points during each of the two bioreactor experiments were thawed at room temperature before DNA extraction. For DNA extraction, the DNeasy PowerSoil Kit (Qiagen) was used according to the kit protocol with two modifications. First, the thawed, liquid bioreactor samples (250 uL) were added directly to PowerBead tubes (Qiagen)

and second, DNA was eluted in 50 µl DEPC-treated water. The DNA concentration was measured using the Qubit dsDNA HS Assay Kit and Qubit 2.0 Fluorometer (Invitrogen, Thermo Fisher Scientific, Oregon, USA). For RNA extraction, used the RNAeasy micro kit as per the kit instructions (Purification of Total RNA from Animal and Human Cells) with the addition of DTT (1.5 uL) and the reduced volume (75 uL) of RTL buffer. After the addition of DTT and RTL buffer, the sample was further disrupted in a PowerBead tube by vertexing for 5 min. No additional DNase step was carried out and the RNA concentration using the TapeStation (Agilent) with the High Sensitivity RNA ScreenTape.

Amplicon sequencing and analysis of RUSITEC samples

Library generation and sequencing was performed at the Vienna Biocentre Core Facility (VBCF). Amplicons were obtained using V3–V4 region of the 16S rRNA gene using primers 341f (5′-TCGTCGGCAGCGTCAGATGTGTATAAGAGACAG) and the reverse primer 785r (5′-GTCTCGTGGGCTCGGAGATGTGTATAAGAGACAG), using PCR conditions described previously[28]. In total, 36 samples from 3 time-points were sequenced on an Illumina MiSeq sequencing platform using a 300 bp paired-end read protocol. The forward reads from the dataset were processed into amplicon sequence variants (ASV) using DADA2 (version 1.9.1, implemented in QIIME2 using denoise-single and trimming from base 20 to 280, otherwise default settings) within the QIIME2 (version 2019.1) environment. Taxonomic assignment was conducted using the RDP package within R (version 1.20.0).

Metagenomic analysis of RUSITEC samples

For metagenomes, paired-end libraries were prepared using the Westburg NGS DNA Library Prep[29], barcoded via PCR, and metagenomic sequencing was done on an Illumina Novoseq 6000 instrument with a 250 bp read length at the Vienna Biocenter Core Facility. The reads were trimmed using Trimmomatic (v. 0.39, flags; "-phred33 ILLUMINACLIP:adapter_sequences.fasta:2:30:10:2:keepBothReads LEADING:5 TRAILING:5 MINLEN:36") and mapped against a Bos Taurus reference genome (GCF_000003055.6, BWA-MEM v. 0.7.17, paired-end, default settings) to filter out any reads obtained from the host. The remaining reads were assembled using SPAdes (v. 3.15.2), reads were mapped using BWA-MEM

(v. 0.7.17, paired-end, default settings), and MAGs were generated using Metabat2 (v. 2.12.2, flags; “-m 1500 -v –unbinned”) with a minimum contig size of 1500 bp. The MAGs were assessed for completeness and contamination using checkM (v. 1.1.3, default settings), and then classified using the classify workflow from the genome taxonomy database toolkit (GTDB-Tk, v. 2.1.0, classify_wf on default settings using database r207). Mapped reads were aligned to the MAGs using blastn (v. 2.10.1+, flags; “-max_target_seqs 1 -max_hsps 1) and the reads per kilobase (kB) were calculated in R (v. 3.6.3).

Analytical Chemistry of RUSITEC experiments

HPLC with refractive index detection (RID) was used to generate the data shown in Supplementary Figure 8A (acetic, propionic, butyric, valeric, and iso-valeric acid). To start, 20 µL of Carrez I solution was added to 300 µL of thawed supernatant, and the mixture was vortexed. Then, after adding 20 µL of Carrez II solution, the sample was vortexed, incubated at room temperature for 10 min, and centrifuged (16,600 x g for 30 min). Approximately 900 µL of the resulting supernatant were filtered using a 0.2 um filter and aliquoted into an HPLC vial (Macherey-Nagel). Analysis was performed on an HPLC system (Agilent 1100 Series Poseidon) equipped with RID, a guard column (Polyspher® OA KC, Merck), and an organic acids column (ICSep ION-300, Transgenomic). The mobile phase, composed of 5 mM sufuric acid, flowed at 0.325 ml/min. The column temperature was maintained at 45°C, the RID was operated at 50°C, and a 40 µL injection volume was used.

GC-MS was also used to generate the data in Supplementary Figure 8B (acetic, propionic, butyric, valeric, iso-valeric, hexanoic, and heptanoic acid). Derivatization with isobutyl chloroformate was followed by liquid–liquid extraction with hexane, and separation and analysis using a Shimadzu GC 2010 gas chromatograph coupled with a Shimadzu TQ-8050 tandem mass spectrometer (Shimadzu), as described previously[30] with modifications. The GC analysis was performed on a Restek Rxi-5Sil column (length 30 m, id 0.25 mm, film thickness 0.25 µm) with the following settings: carrier gas helium (minimum purity 99.9995%) in constant linear velocity mode at 30 cm/sec, SSL Injector at 250 °C, septum purge at 6 mL/ min, and an injection volume of 1 µL. The GC oven temperature program for acetic, propionic, butyric, isovaleric, valeric,

hexanoic acid was as follows: 50 °C for 3.27 min, ramp 20 °C/min to 162 °C, and then 40 °C/min until 290 °C (held for 3 min) with a split ratio of 1:60, whereas the GC oven temperature program for heptanoic, octanoic, and lactic acid was set to the following: 100 °C for 1.00 min, ramp 15 °C/min to 197 °C, then 30 °C/min until 252 °C, then 40 °C/min until 290 °C (held for 3 min) with a split ratio of 1:3. The mass spectrometer was operated in SIM mode with electron impact ionization at 70 eV, MS transfer line temperature at 250 °C, and MS source temperature at 200 °C. Recoveries of the organic acids were corrected by the use of the corresponding labelled compounds acetic,2-2-2-D3, propionic-D5, butyric-D7, isovaleric-D7, valeric-D9, hexanoic-D11, heptanoic-D13, octanoic acid-D15, and sodium L-lactate-3,3,3,-D3 acid), which were added before the derivatization procedure.

For analysis of DON, ZEN and their associated metabolites (de-epoxy deoxynivalenol, DOM; α- and β-zearalenol, α- and β-ZEL, Supplementary Figure 6), 200 µL of the bioreactor sample was mixed with 5 µL of acetic acid (VWR) and 600 µL of ethyl acetate (ChemLab). The mixture was shaken for 10 min on an overhead shaker and subsequently centrifuged (5 min, 19000 x *g*). The organic phase was transferred into a 2 mL reaction tube. The extraction procedure of the initial sample was then repeated, and the two supernatants combined. Pooled organic phases were dried (Concentrator 5301, Eppendorf), reconstituted in 200 µL acetonitrile/water/formic acid (v/v/v, 20/79/1) and clarified by centrifugation (10 min, 19000 x *g*) for LC-MS/MS analysis, which was performed using a 1290 Infinity High-Performance Liquid Chromatography (HPLC) system (Agilent Technologies) coupled to a 5500 QTrap mass spectrometer equipped with an electrospray ionization source (SCIEX) as described previously[31] with minor modifications. Chromatographic separation (flow rate 650 µL/min, injection volume 1 µL) was achieved by increasing mobile phase B from initially 0% (0.1 min) to 30% (2.2 min), 55% (5.8 min) and finally 100% (5.9 min). This proportion of phase B was kept until 6.4 min, subsequently decreased to 0% (6.5 min) and held until the end of the method (7.5 min). Mass spectrometric detection of DON and DOM was carried out in multiple reaction monitoring mode with negative polarity using the following m/z transitions: DON quant 355.1/59.0 Da, declustering potential (DP) -45 V, entrance potential (EP) -10 V, collision energy (CE) -50 V, collision cell exit potential (CXP) -9 V; DON qual 355.1/265.0 Da, DP -45 V, EP -10 V, CE -22 V, CXP -19 V; DOM quant 339.1/59.1 Da, DP -70 V, EP -10 V, CE -42 V, CXP -7V; DOM qual 339.1/249.0 Da,

DP -70 V, EP -10 V, CE -14 V, CXP -15 V. The limit of quantification for DON and DOM was 2.5 µg/L, respectively.

Supplementary Discussion

Genome-wide sweeps and co-existence

After showing that both *Megasphaera* species are particularly abundant compared to other putative chain elongators in the calf rumen, we observed that their genomic diversity is consistent with genome-wide sweeps. Such sweeps occur when an adaptation leads to a single genome, by way of hitchhiking, replacing all others within its niche. This is then followed by re-diversification, often via recombination. We recently observed genome-wide sweeps in relatively closely related populations of *Campylobacter* co-existing in the rumen[32]. These likely split from a common ancestor relatively recently. In contrast, *M. elsdenii* and *M. hexanoica* are far more divergent and, considering their shared dynamics across multiple mammalian host species, have likely been stably co-existing for millions of years. Also unlike the rumen Campylobacter, we did not observe any closely related, co-existing populations within the two *Megasphaera* species. Thus, even though several general traits seem to be shared between *M. elsdenii* and *M. hexanoica*, they are expected, based on their level of genomic divergence, to be highly optimized to unique niches that have enabled their long-term, stable co-existence. According to theory, such co-existence is driven by trade-offs and, in the case of a shared, variable resource, such as lactate, niche optimization has likely involved adaptation to resource fluctuations[33,34].

Study limitations

One of the limitations of this study is that we only focused on the rumen. However, our two focal *Megasphaerea* species were observed to co-exist in both the rumen and the swine gut. It would thus be interesting to understand what host-specific adaptations are present in *M. elsdenii* or *M. hexanoica* populations despite the generally low within-species diversity. The general findings for the specific lactate-utilization strategies are very likely conserved across strains isolated from

both hosts (ruminants and swine). Outside of lactate utilization, however, it very possible that strains belonging to the same *Megasphaera* species from different hosts are differentiated, for example, in their sugar-utilizing abilities and extent of catabolic repression. This may indeed be expected as a consequence of substantial differences in commercial feeding regimens.

In this study, we did not confirm a specific lactate-based co-existence mechanism, such as a gleaner opportunist trade-off[35,36], for *M. elsdenii* and *M. hexanoica*. This would have been supported by *M. hexanoica* growing more rapidly than *M. elsdenii* at low lactate concentrations, making *M. hexanoica* the gleaner. The higher yield strategy by *M. hexanoica* may outcompete faster growth when competition is minimal[37]. Indeed, this has been demonstrated in yeast, where resource competition is limited by spatial structure[38].

Supplementary References

1. Parks, D. H. *et al.* GTDB: an ongoing census of bacterial and archaeal diversity through a phylogenetically consistent, rank normalized and complete genome-based taxonomy. *Nucleic Acids Res* **50**, D785–D794 (2022).
2. Furman, O. *et al.* Stochasticity constrained by deterministic effects of diet and age drive rumen microbiome assembly dynamics. *Nat Commun* **11**, 1904 (2020).
3. Malmuthuge, N., Liang, G. & Guan, L. L. Regulation of rumen development in neonatal ruminants through microbial metagenomes and host transcriptomes. *Genome Biol* **20**, 172 (2019).
4. Bankevich, A. *et al.* SPAdes: A New Genome Assembly Algorithm and Its Applications to Single-Cell Sequencing. *Journal of Computational Biology* **19**, 455–477 (2012).
5. Li, H. Aligning sequence reads, clone sequences and assembly contigs with BWA-MEM. (2013).
6. Kang, D. D. *et al.* MetaBAT 2: an adaptive binning algorithm for robust and efficient genome reconstruction from metagenome assemblies. *PeerJ* **7**, e7359 (2019).
7. Stewart, R. D. *et al.* Compendium of 4,941 rumen metagenome-assembled genomes for rumen microbiome biology and enzyme discovery. *Nat Biotechnol* **37**, 953–961 (2019).
8. Jain, C., Rodriguez-R, L. M., Phillippy, A. M., Konstantinidis, K. T. & Aluru, S. High throughput ANI analysis of 90K prokaryotic genomes reveals clear species boundaries. *Nat Commun* **9**, 5114 (2018).
9. Parks, D. H., Imelfort, M., Skennerton, C. T., Hugenholtz, P. & Tyson, G. W. CheckM: assessing the quality of microbial genomes recovered from isolates, single cells, and metagenomes. *Genome Res* **25**, 1043–1055 (2015).
10. Minh, B. Q. *et al.* IQ-TREE 2: New Models and Efficient Methods for Phylogenetic Inference in the Genomic Era. *Mol Biol Evol* **37**, 1530–1534 (2020).

11. Cantalapiedra, C. P., Hernández-Plaza, A., Letunic, I., Bork, P. & Huerta-Cepas, J. eggNOG-mapper v2: Functional Annotation, Orthology Assignments, and Domain Prediction at the Metagenomic Scale. *Mol Biol Evol* **38**, 5825–5829 (2021).
12. Annie Yu, X. *et al.* Genome-wide sweeps create fundamental ecological units in the human gut microbiome 2. doi:10.1101/2024.05.25.595854.
13. Brown, C. T., Olm, M. R., Thomas, B. C. & Banfield, J. F. Measurement of bacterial replication rates in microbial communities. *Nat Biotechnol* **34**, 1256–1263 (2016).
14. O'Hara, E. *et al.* Investigating temporal microbial dynamics in the rumen of beef calves raised on two farms during early life. *FEMS Microbiol Ecol* **96**, (2020).
15. Kodithuwakku, H. *et al.* Alterations in rumen microbiota via oral fiber administration during early life in dairy cows. *Sci Rep* **12**, 10798 (2022).
16. Gaire, T. N. *et al.* Age influences the temporal dynamics of microbiome and antimicrobial resistance genes among fecal bacteria in a cohort of production pigs. *Anim Microbiome* **5**, 2 (2023).
17. Wang, X. *et al.* Longitudinal investigation of the swine gut microbiome from birth to market reveals stage and growth performance associated bacteria. *Microbiome* **7**, 109 (2019).
18. McGovern, E. *et al.* Investigation into the effect of divergent feed efficiency phenotype on the bovine rumen microbiota across diet and breed. *Sci Rep* **10**, 15317 (2020).
19. Kamke, J. *et al.* Rumen metagenome and metatranscriptome analyses of low methane yield sheep reveals a Sharpea-enriched microbiome characterised by lactic acid formation and utilisation. *Microbiome* **4**, (2016).
20. Stepanchenko, N. *et al.* Microbial composition, rumen fermentation parameters, enteric methane emissions, and lactational performance of phenotypically high and low methane-emitting dairy cows. *J Dairy Sci* **106**, 6146–6170 (2023).
21. Dill-McFarland, K. A., Breaker, J. D. & Suen, G. Microbial succession in the gastrointestinal tract of dairy cows from 2 weeks to first lactation. *Sci Rep* **7**, 40864 (2017).
22. Callahan, B. J. *et al.* DADA2: High-resolution sample inference from Illumina amplicon data. *Nat Methods* **13**, 581–583 (2016).
23. Park, T., Cersosimo, L. M., Radloff, W., Zanton, G. I. & Li, W. The rumen liquid metatranscriptome of post-weaned dairy calves differed by pre-weaning ruminal administration of differentially-enriched, rumen-derived inocula. *Anim Microbiome* **4**, 4 (2022).
24. Seemann, T. Prokka: rapid prokaryotic genome annotation. *Bioinformatics* **30**, 2068–2069 (2014).
25. Putri, G. H., Anders, S., Pyl, P. T., Pimanda, J. E. & Zanini, F. Analysing high-throughput sequencing data in Python with HTSeq 2.0. *Bioinformatics* **38**, 2943–2945 (2022).
26. Candry, P. *et al.* A novel high-throughput method for kinetic characterisation of anaerobic bioproduction strains, applied to Clostridium kluyveri. *Sci Rep* **8**, 9724 (2018).
27. McDougall, E. I. Studies on ruminant saliva. 1. The composition and output of sheep's saliva. *Biochem J* **43**, 99–109 (1948).
28. Klindworth, A. *et al.* Evaluation of general 16S ribosomal RNA gene PCR primers for classical and next-generation sequencing-based diversity studies. *Nucleic Acids Res* **41**, e1–e1 (2013).

29. Sims, J., Sestini, G., Elgert, C., von Haeseler, A. & Schlögelhofer, P. Sequencing of the Arabidopsis NOR2 reveals its distinct organization and tissue-specific rRNA ribosomal variants. *Nat Commun* **12**, 387 (2021).
30. Ueyama, J. *et al.* Freeze-drying enables homogeneous and stable sample preparation for determination of fecal short-chain fatty acids. *Anal Biochem* **589**, 113508 (2020).
31. Gruber-Dorninger, C. *et al.* Metabolism of Zearalenone in the Rumen of Dairy Cows with and without Application of a Zearalenone-Degrading Enzyme. *Toxins (Basel)* **13**, 84 (2021).
32. Strachan, C. R. *et al.* Differential carbon utilization enables co-existence of recently speciated Campylobacteraceae in the cow rumen epithelial microbiome. *Nat Microbiol* **8**, 309–320 (2023).
33. Tilman, D. *Resource Competition and Community Structure.* vol. 17 (Princeton University Press, 1982).
34. Chesson, P. Mechanisms of Maintenance of Species Diversity. *Annu Rev Ecol Syst* **31**, 343–366 (2000).
35. Yamamichi, M. & Letten, A. D. Extending the gleaner–opportunist trade-off. *Journal of Animal Ecology* **91**, 2163–2170 (2022).
36. Matin, A. & Veldkamp, H. Physiological Basis of the Selective Advantage of a Spirillum sp. in a Carbon-limited Environment. *J Gen Microbiol* **105**, 187–197 (1978).
37. Pfeiffer, T. & Bonhoeffer, S. Evolutionary Consequences of Tradeoffs between Yield and Rate of ATP Production. *Zeitschrift für Physikalische Chemie* **216**, (2002).
38. Bachmann, H. *et al.* Availability of public goods shapes the evolution of competing metabolic strategies. *Proceedings of the National Academy of Sciences* **110**, 14302–14307 (2013).

Supplementary Figures

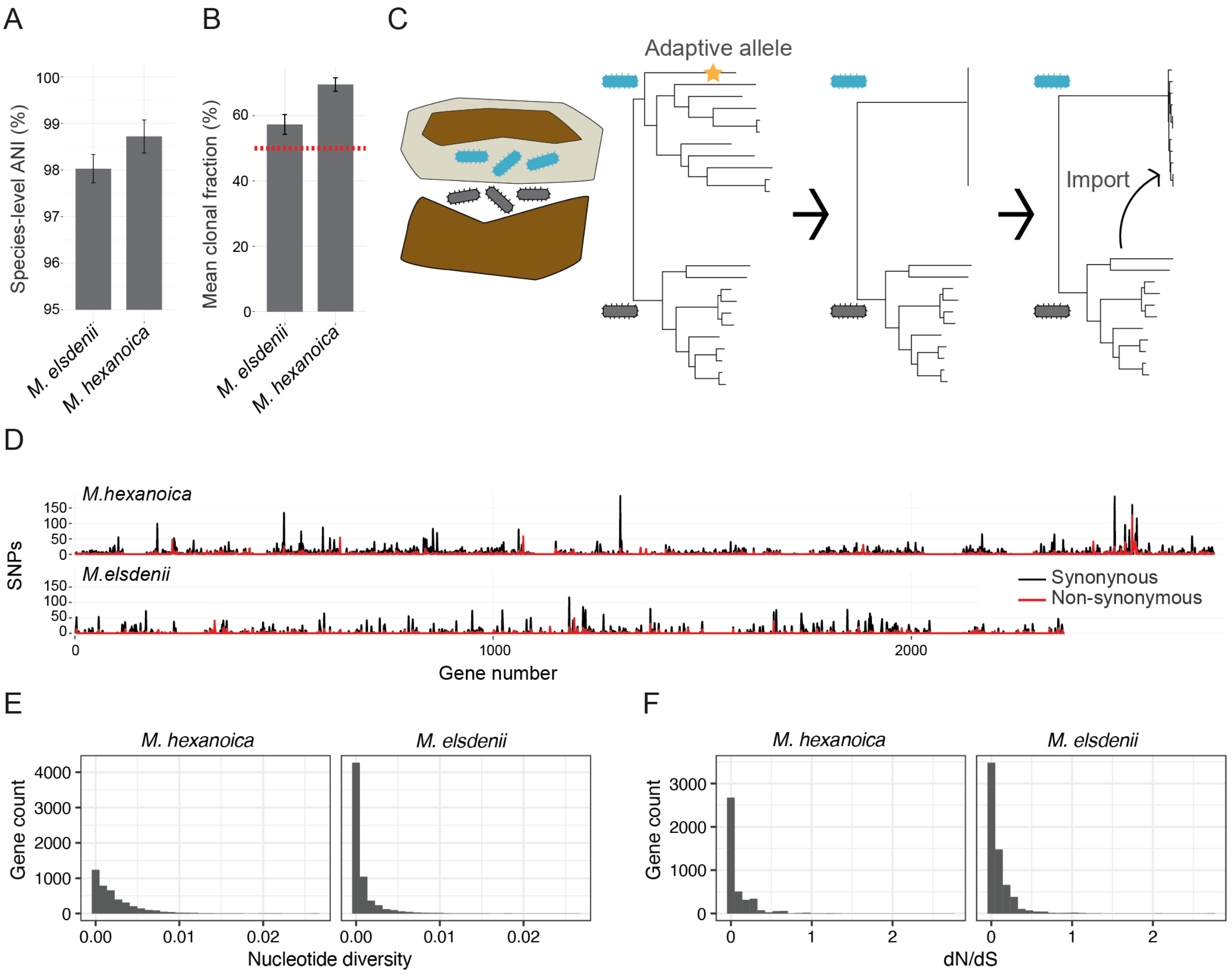

Supplementary Figure 1. Evidence for genome-wide sweeps within the *Megasphaera*. **A)** The species-level ANI was calculated pairwise between the genomes and MAGs from those in the latest release of the genome taxonomy database (GTDB, v. 226.0)[1]. Only those genomes with >90% completeness and <1% contamination were used (n=47 and n=11 for *M. elsdenii* and *M. hexanoica*, respectively). Note that in the GTDB *M. hexanoica* was renamed to *Caecibacter massiliensis*. The mean of the pairwise comparisons is shown and the error bars represent the standard deviation. **B)** The estimated clonal fraction using MAGs and genomes that are used in Figure 2A. The red line denotes 50% and, thus, where the majority of the genome is designated as clonal. **C)** Graphical overview of the process of genome-wide sweeps and consequences for genome diversity. Here, the light blue bacterial population inhabits a unique niche. After a genome acquires an adaptive allele that is under selection, it outcompetes its next of kin, thus purging genome-wide diversity. This is because, in genome-wide sweeps, the entire genome hitchhikes along with the adaptive allele. After the genome-wide sweep, genomes re-diversify at specific regions by the import of alleles via horizontal gene transfer (HGT). Thus, a pattern of low genome-wide diversity interrupted by local spikes within a population is consistent with a genome-wide sweep. **D)** The SNPs in genes were detected in the calf metagenomes. These are shown across the two *Megasphaera* genomes and separated into synonymous and non-synonymous SNPs. The same SNPs were used to generate the distributions in **E)** and **F)**.

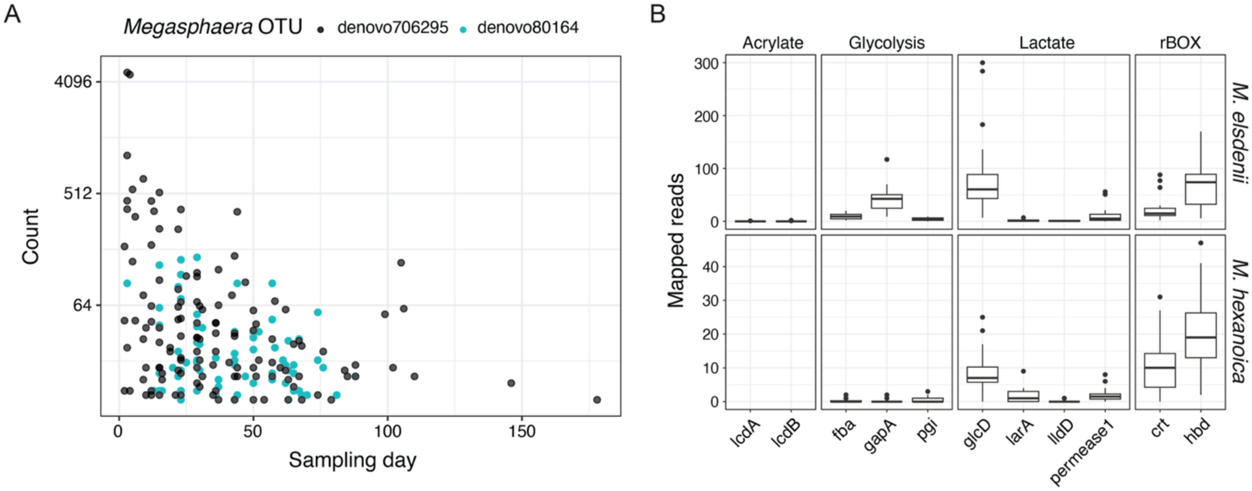

Supplementary Figure 2. A) Core successional operational taxonomic units (OTUs) from Furman *et al.* 2020 that were assigned to the genera *Megasphaera*[2]. Only those OTUs that were detected over 10 times in more than 3 samples are shown. **B)** Raw expression values for genes in addition to those shown in Figure 3C. The top and bottom panels show genes mapped to *M. elsdenii* and *M. hexanoica*, respectively. Marker genes are grouped as either belonging to the acrylate pathway, glycolysis, lactate utilization (excluding the acrylate pathway), and reverse beta-oxidation (rBOX).

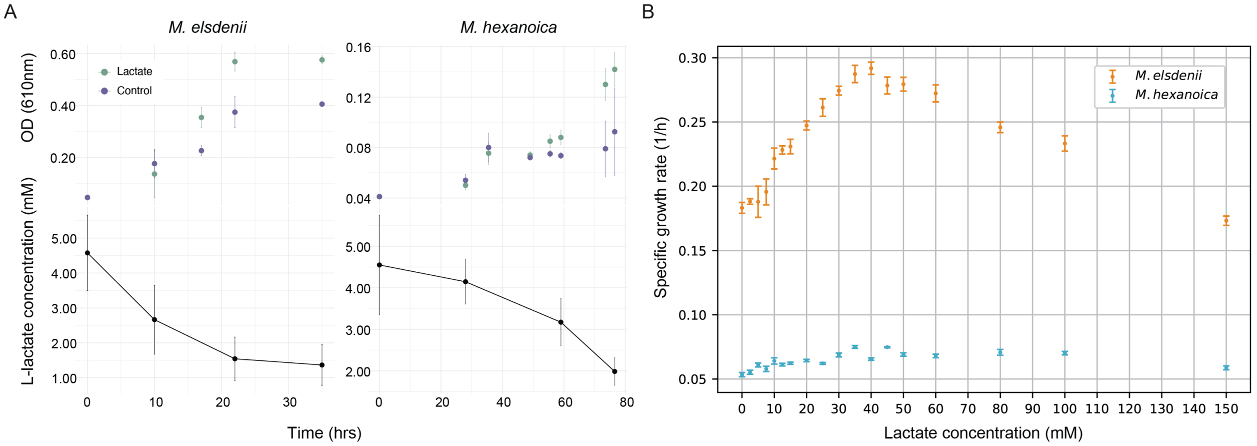

Supplementary Figure 3. A) The two strains, *M. elsdenii* DSM20460 and *M. hexanoica* MH, were grown in rich media with and without the presence of lactate. In all other *in vitro* growth experiments, *M. hexanoica* DSM106893 was used. The median OD and lactate concentration are shown for time points sampled from Hungate tubes throughout the growth curve. Error bars represent the standard deviation (n=6 biological replicates). **B)** Maximum growth rates derived from high-throughput growth experiments for both *M. elsdenii* (DSM20460) and *M. hexanoica* (DSM106893) with lactate as the main electron donor. Lactate concentration on the X-axis denotes the starting lactate concentration in the media. Relatively slow growth was observed in lactate-free media (μ = ~0.17 h^{-1} for *M. elsdenii* and ~0.05 h^{-1} for *M. hexanoica*) in the rich basal medium. The concentrations of lactate that yield a midpoint growth rate between m at 0 mM and μ_{max} are achieved at approximately 20 mM for *M. elsdenii* and 10 mM for *M. hexanoica*, indicating that the latter species may have a higher affinity for lactate. The mean is shown with error bars representing standard deviation (n=3 biological replicates).

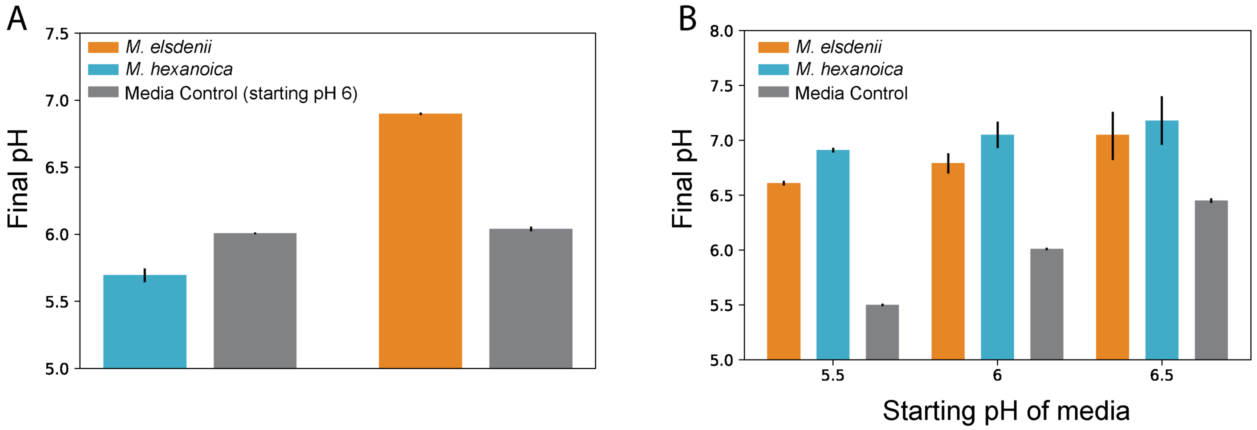

Supplementary Figure 4. Modification of media pH by *M. elsdenii* and *M. hexanoica*. **A)** Control media and final media pH from the growth data collected for Figure 4 where both lactate and glucose were added as electron donors. **B)** Control media and final pH across different starting pH values from the data collected in Figure 6 where only lactate was the main electron donor.

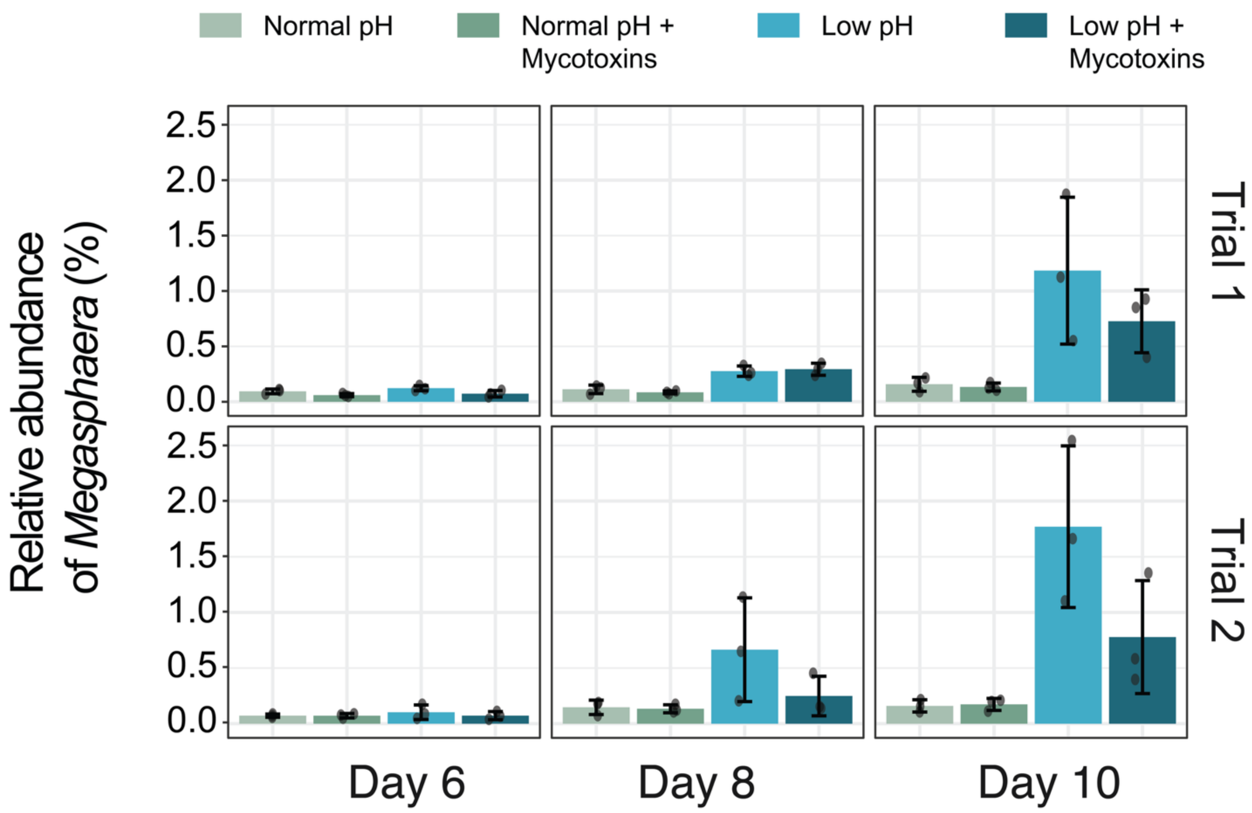

Supplementary Figure 5. *M. hexanoica* amplicon dynamics in the rumen simulation technique (RUSITEC) experiments. Mean relative abundance of a single ASV classified as *M. hexanoica* from both replicated bioreactor experiments. Error bars show the standard deviation (n = 3).

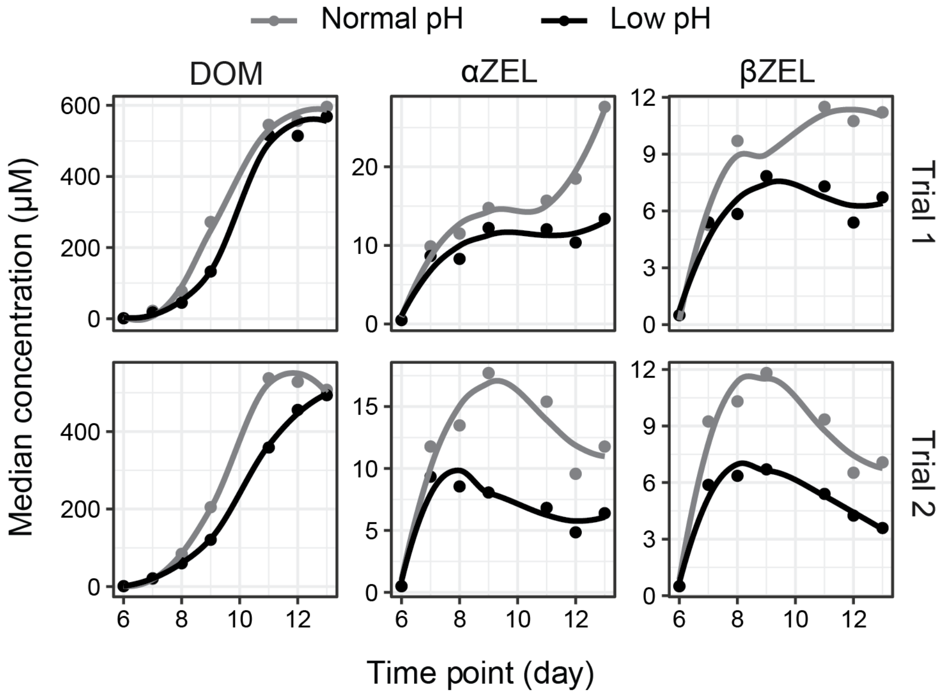

Supplementary Figure 6. Mycotoxin modification in the rumen simulation technique (RUSITEC) experiments. Metabolites of DON (DOM, de-epoxy deoxynivalenol) and ZEN (α- and β-ZEL, α-zearalenol, β-zearalenol) as determined by LC-MS/MS. The median is shown for each treatment (n=3) in which the mycotoxin-containing fungal extract was added. Replicated experiments are shown in the top and bottom panel.

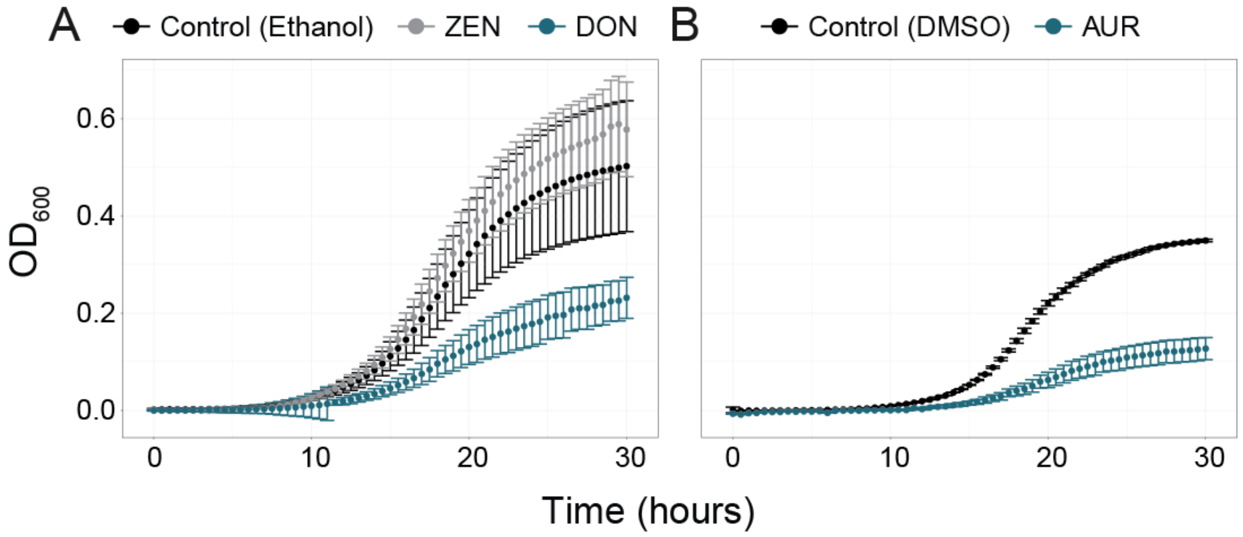

Supplementary Figure 7. Pure mycotoxins directly inhibit *M. hexanoica*. The representative strain of *M. hexanoica* was grown with and without the presence of 35 µM of pure mycotoxins. A) The results for deoxynivalenol (DON) and zearalenone (ZEN), which were soluble in ethanol and therefore compared to an ethanol control. **B)** The results for aurofusarin (AUR), which was soluble in DMSO and compared to a DMSO control. The mean OD is shown, and error bars represent the standard deviation (n = 6).

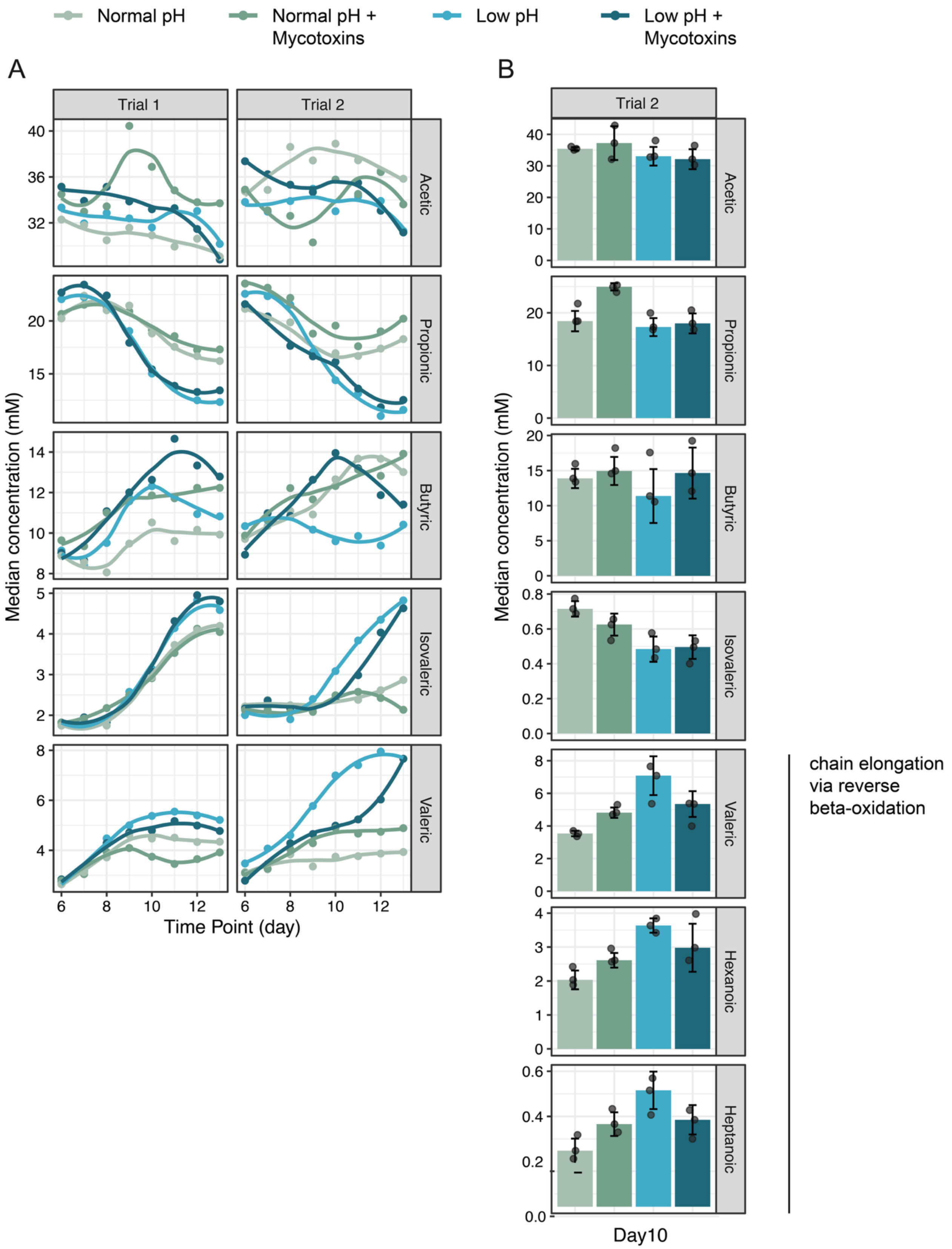

Normal pH
Normal pH + Mycotoxins
Low pH
Low pH + Mycotoxins
A
B
Trial 1
Trial 2
Acetic
Propionic
Butyric
Isovaleric
Valeric
Hexanoic
Heptanoic
Median concentration (mM)
Time Point (day)
Day10
chain elongation via reverse beta-oxidation

Supplementary Figure 8. Organic acid production in the rumen simulation technique (RUSITEC) experiments. **A)** Organic acids measured by HPLC where the median is shown for each treatment (n=3). The replicated experiments are shown side by side. **B)** Organic acids measured by GC-MS from experimental day 10 of the second replicated experiment. The median (n=3) is shown with error bars representing the standard deviation. Lactate was also measured but not detected.

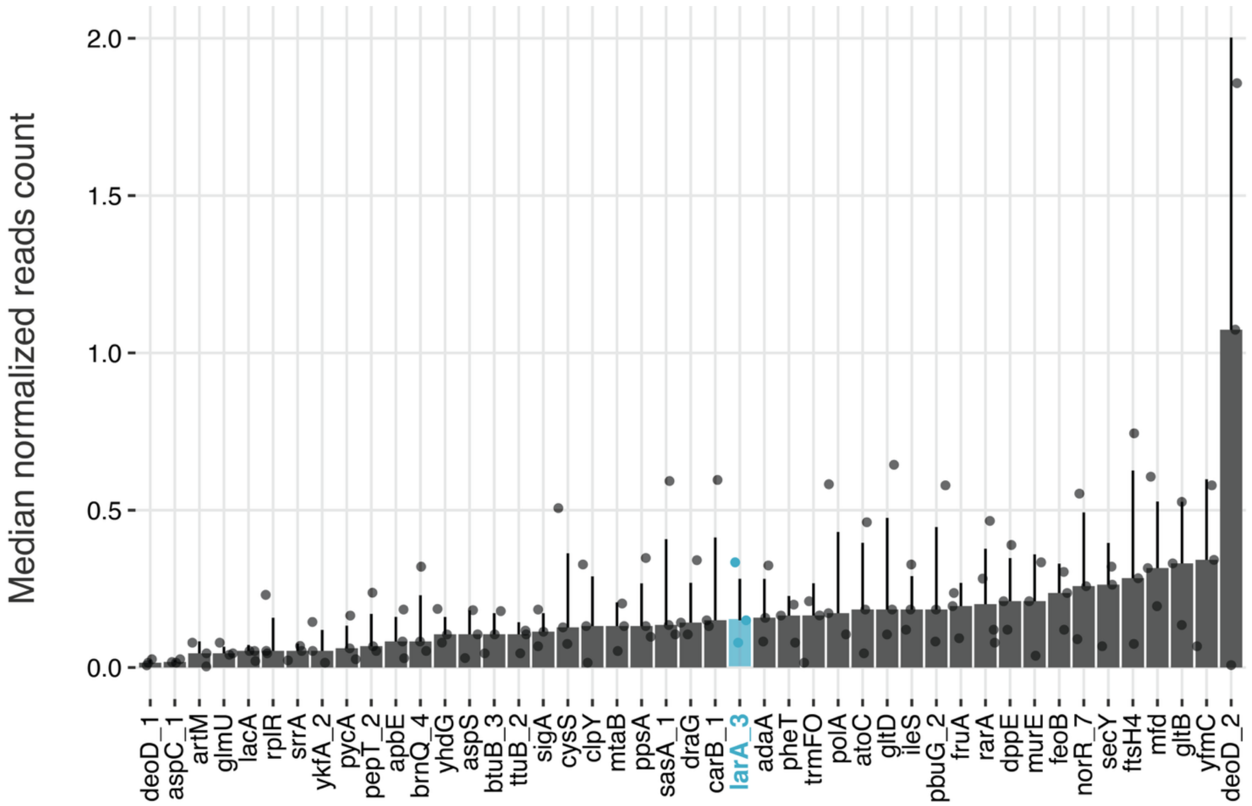

Supplementary Figure 9. Top expressed genes by *M. hexanoica* in the rumen simulation technique (RUSITEC) experiment. Metatranscriptomes were collected from experimental day 10. Reads were normalized by the total number of reads that were mapped to *M. hexanoica,* and genes were ordered by median expression level from right to left.

Legends for Supplementary Tables

Supplementary Table 1. Overview of genomes of metagenome-assembled genomes analyzed, including acrylate pathway annotations and GTDB classification.

Supplementary Table 2. Number of 16S rRNA operons in rumen *Megasphaera* genomes and inferred microbial population replication rates.

Supplementary Table 3. Classification and quality of *M. hexanoica* MAG recovered from the RUSITEC.

Supplementary Table 4. GTDB classification of MAGs revered from the RUSITEC.

Supplementary Table 5. Most abundant amplicons in RUSITEC experiments.

Supplementary Table 6. RUSITEC feed and buffer composition.
